# Glycan complexity dictates microbial resource allocation in the large intestine

**DOI:** 10.1038/ncomms8481

**Published:** 2015-06-26

**Authors:** Artur Rogowski, Jonathon A. Briggs, Jennifer C. Mortimer, Theodora Tryfona, Nicolas Terrapon, Elisabeth C. Lowe, Arnaud Baslé, Carl Morland, Alison M. Day, Hongjun Zheng, Theresa E. Rogers, Paul Thompson, Alastair R. Hawkins, Madhav P. Yadav, Bernard Henrissat, Eric C. Martens, Paul Dupree, Harry J. Gilbert, David N. Bolam

**Affiliations:** 1 Institute for Cell and Molecular Biosciences, The Medical School, Newcastle University, Newcastle upon Tyne NE2 4HH, UK; 2 Department of Biochemistry, University of Cambridge, Tennis Court Road, Cambridge CB2 1QW, UK; 3 Architecture et Fonction des Macromolécules Biologiques (AFMB), UMR 7257 CNRS, Université Aix-Marseille, 163 Avenue de Luminy, 13288 Marseille, France; 4 Department of Microbiology and Immunology, University of Michigan Medical School, Ann Arbor, Michigan 48109, USA; 5 United States Department of Agriculture- Agricultural Research Service, Eastern Regional Research Center, 600 East Mermaid Lane, Wyndmoor, Pennsylvania 19038, USA; 6 Department of Biological Sciences, King Abdulaziz University, Jeddah, Saudi Arabia

## Abstract

The structure of the human gut microbiota is controlled primarily through the degradation of complex dietary carbohydrates, but the extent to which carbohydrate breakdown products are shared between members of the microbiota is unclear. We show here, using xylan as a model, that sharing the breakdown products of complex carbohydrates by key members of the microbiota, such as *Bacteroides ovatus*, is dependent on the complexity of the target glycan. Characterization of the extensive xylan degrading apparatus expressed by *B. ovatus* reveals that the breakdown of the polysaccharide by the human gut microbiota is significantly more complex than previous models suggested, which were based on the deconstruction of xylans containing limited monosaccharide side chains. Our report presents a highly complex and dynamic xylan degrading apparatus that is fine-tuned to recognize the different forms of the polysaccharide presented to the human gut microbiota.

## Abstract (web summary)

The human gut microbiota helps us to degrade complex dietary carbohydrates such as xylan and, in turn, the carbohydrate breakdown products control the structure of the microbiota. Here the authors characterize the xylan-degrading apparatus of a key member of the gut microbiota, *Bacteroides ovatus*.

## Graphical abstract

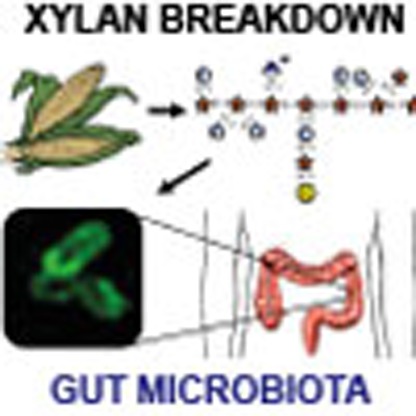

Genomic and metagenomic data show that the catabolism of complex dietary carbohydrates (CDCs), also known as dietary fibre, play a central role in defining the structure of the human gut microbiota (HGM)^1^,^2^. The Bacteroidetes of the HGM, which include the genus *Bacteroides*, are viewed as glycan-degrading generalists capable of utilizing an extensive array of both dietary (plant and animal) and host complex carbohydrates^3^,^4^. The other main phyla of the gut, Firmicutes and Actinobacteria (particularly *Bifidobacterium* spp.), also have the capacity to degrade glycans, but appear more specialized with a preference for plant storage polysaccharides (starch and fructan) and oligosaccharides, such as those derived from arabinoxylans (AXs)^5^,^6^. Current strategies deployed to manipulate the composition of the HGM to maximize human health are centred on the use of complex carbohydrates (prebiotics)^7^ to encourage the expansion of beneficial (probiotic) microorganisms^8^. The effects of these strategies are ultimately dependent on the glycan food web in the HGM^9^,^10^. However, the extent to which members of the HGM act in synergy to degrade and utilize CDCs is currently unclear. Furthermore, while genomic and metagenomic sequencing has identified an extensive array of putative glycan degrading enzymes encoded by the HGM^1^,^11^,^12^, the lack of functional signposts, obtained through enzyme characterization, greatly restricts our capacity to identify the activities displayed by these candidate glycanases and thus predict the glycan preferences of specific members of the HGM.

Xylan, a major component of many plant cell walls, is one of the most variable plant structural polysaccharides, and is a common component of cereal-derived human foods^13^,^14^,^15^. The main classes of xylan are the glucuronoxylans (GXs), arabinoxylans (AXs) and glucuronoarabinoxylans (GAXs). The designation of these three polysaccharides reflects the nature of the monosaccharides that decorate the conserved β1,4-xylose (Xyl) backbone (Fig. 1). Many GXs and AXs are relatively simple. In contrast, the number and complexity of the side chains on cereal GAXs, exemplified by corn bran xylan (CX) (Fig. 1) explains why these glycans are recalcitrant to breakdown by known xylan-degrading enzyme systems^13^.

*Bacteroides ovatus*, a prominent member of the HGM, was previously shown to utilize an extensive range of plant polysaccharides, including xylans^4^. Here we explored the mechanisms by which this bacterium degrades the different forms of the polysaccharide presented to the HGM. Our data show that *B. ovatus* has evolved a highly dynamic xylan degrading system that can respond to the different forms of the xylose polymer. The bacterium has recruited several enzymes from glycoside hydrolase (GH) families^16^ not previously associated with xylan degradation. We also show that, although polysaccharide breakdown products (PBPs) are released during degradation of both simple and complex xylans by *B. ovatus*, the ability of other members of the HGM to use these PBPs is dictated by the complexity of the target xylan.

## Results

### *B. ovatus* contains two discrete loci activated by xylans

The genomes of Bacteroidetes contain numerous polysaccharide utilization loci (PULs) that are optimized to orchestrate the degradation of specific polysaccharides^4^,^17^,^18^,^19^. The glycans targeted by the PULs activate transcription of their cognate loci. *Bacteroides ovatus* strain ATCC 8483 contains two PULs, spanning locus tags *bacova_03417-50* (large xylan PUL or PUL-XylL) and *bacova_04385-94* (small xylan PUL or PUL-XylS), that are activated when the organism is grown on wheat arabinoxylan^4^ (WAX) (Fig. 2a,b). In contrast, linear xylooligosaccharides (preferentially xylotetraose) induce transcription of PUL-XylS alone^4^. Here we show that growth on CX leads to significant activation of PUL-XylL and only very low activation of PUL-XylS, while glucuronoxylan (from birch; BGX) activates only PUL-XylS (Fig. 2c). These data suggest that the degradative system encoded by PUL-XylS targets simple linear or glucurono-substituted xylans, while the products of PUL-XylL drive the metabolism of more complex decorated forms of the hemicellulose. Bacteroidetes spp. are known to express enzymes capable of breaking down simple forms of xylan^20^,^21^; however, the mechanisms by which these microorganisms utilize complex cereal GAXs (for example, CX) are not known. Both PULs encode glycanases belonging to GH families^16^ that are known to contribute to the degradation of simple xylans (GH3, GH10, GH30, GH43, GH67 and GH115)^22^ (Fig. 2a,b). Significantly, PUL-XylL also directs the synthesis of enzymes belonging to families (GH31, GH95, GH97 and GH98)^4^ not previously implicated in the deconstruction of the hemicellulose (Fig. 2a).

### Surface acting xylan-degrading enzymes

Bioinformatic analysis, in conjunction with cell localization studies and whole-cell assays, was used to identify the enzymes encoded by PUL-XylS and PUL-XylL that are displayed on the bacterial surface. The presence of a predicted Type II lipoprotein signal sequence or uncleaved transmembrane (TM) anchor in seven GH family proteins indicated that they were membrane attached. The cell localization of these seven proteins was evaluated experimentally by (i) fluorescence microscopy of whole cells treated with labelled antibodies directed against the appropriate proteins and (ii) western blotting after removal of surface proteins with Proteinase K. The data indicated that BACOVA_03419 (GH3), BACOVA_03421 (GH43), BACOVA_03431 (inactive GH10, see later results), BACOVA_03432 (GH30), BACOVA_03433 (GH98) and BACOVA_04390 (GH10) were presented on the surface of *B. ovatus* (Fig. 3). The only lipoprotein facing the periplasm was the GH43 enzyme BACOVA_03425. These conclusions were further supported by whole-cell assays of the *B. ovatus* mutant lacking PUL-XylS (ΔPUL-XylS) incubated with BGX. The product profile of ΔPUL-XylS was extremely similar to that of recombinant BACOVA_03432 GH30 against the polysaccharide (Supplementary Fig. 1a). Thus, BGX degradation is mediated predominantly by BACOVA_03432 in the ΔPUL-XylS mutant, confirming the extracellular location of the GH30 enzyme. Whole-cell assays also showed that the PUL-XylL encoded GH43 arabinofuranosidase, BACOVA_03421 (see results below), was extracellular, as arabinose was released from WAX by the ΔPUL-XylS mutant (Supplementary Fig. 1b). It is surprising that BACOVA_03419, a GH3 β-xylosidase, is located on the surface as its obvious role is to contribute to the degradation of the xylooligosaccharides generated in the periplasm (Supplementary Fig. 2a). BACOVA_03419, however, displays only low activity against linear β1,4-xylooligosaccharides, but, unlike the cytoplasmic xylosidase, BACOVA_04386 (see results below), will cleave linkages in which the leaving group xylose is decorated (Supplementary Table 1 and Supplementary Fig. 2b,c). While the roles of BACOVA_03419 and BACOVA_03421 in xylan degradation are unclear, we speculate that the enzymes contribute to the surface ‘trimming’ of complex xylans.

Three of the surface enzymes (BACOVA_04390 GH10, BACOVA_03432 GH30 and BACOVA_03433 GH98 (Fig. 3a,b) exhibited endo activity and therefore are likely involved in cleaving the xylan backbone before importing into the periplasm. Studies on recombinant BACOVA_04390 GH10 showed that the endo-xylanase displayed a catalytic efficiency against xylan ∼30-fold lower than typical GH10 enzymes^23^,^24^ (Supplementary Table 2). The limit products against sparsely decorated xylan contains oligosaccharides with a degree of polymerization (d.p.) >4 (Supplementary Fig. 3a). Typical GH10 xylanases^23^,^24^, exemplified by the periplasmic enzyme BACOVA_04387 generate xylose and xylobiose as the final products (Supplementary Fig. 3b). These data suggest that BACOVA_04390 contains an extended substrate binding cleft that requires a high degree of occupancy for catalysis to occur. Substrate depletion data using xylooligosaccharides confirmed that the enzyme had very poor activity against substrates with a d.p. ≤5 compared with typical GH10 enzymes (Supplementary Table 2). BACOVA_04390 has an unusual primary sequence in which the canonical GH10 (β/α)_8_-fold barrel is interrupted by two copies of a non-catalytic carbohydrate-binding module (CBM) that is distantly related to CBM family 4, suggesting that the two CBMs contribute to an extended substrate binding cleft^21^,^25^. Indeed a recent study has shown that the internal CBMs from a related *B. intestinalis* GH10 bind xylans and long xylooligosaccharides^21^. This view is consistent with the observation that endo-acting enzymes, which specifically target large polymers, contain additional subsite(s) likely compensating for relatively weak binding at subsites proximal to the active site^26^,^27^.

The GH30 enzyme BACOVA_03432 produced a range of large oligosaccharides from BGX (Supplementary Fig. 1a and Supplementary Table 2), but was not active on WAX or CX. This specificity is typical of GH30 glucuronoxylanases and is driven by recognition of the (Me)GlcA side chain at the -2 subsite of these enzymes^28^. While the lack of activity against CX is somewhat surprising as the enzyme is part of PUL-XylL, it may be that less heavily decorated GAXs are the target substrates for this enzyme.

**Figure 1:**
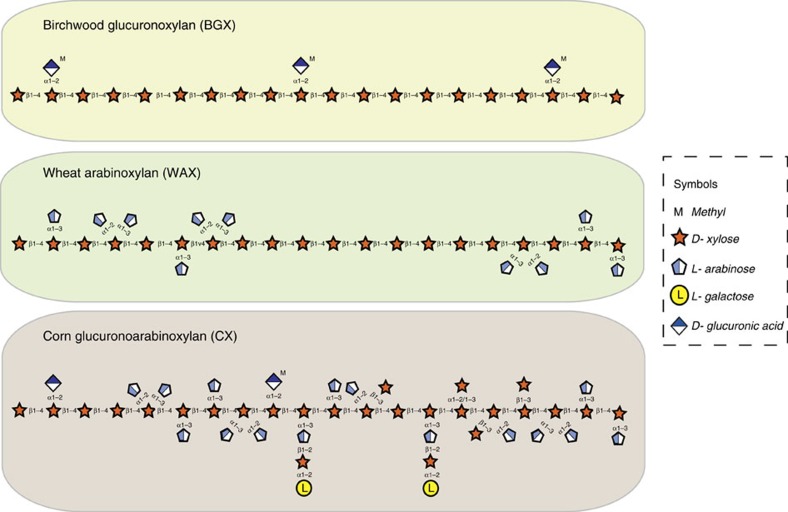
Schematic of the structures of the main classes of xylan. The monosaccharides and linkages in the main classes of xylan are shown and are represented in their Consortium for Functional Glycomics format^69^. The xylans used in this study were from birchwood (birch glucuronoxylan; BGX), wheat flour (wheat arabinoxylan; WAX) and corn bran (corn glucuronoarabinoxylan; CX).

The surface GH98 enzyme BACOVA_03433 also generates very large oligosaccharides but exclusively from CX (Fig. 4a,b). As BACOVA_03433 is a member of GH98, a family that hitherto contained only endo-β-D-galactosidases^29^, it was formally possible that the enzyme targets rare β-D-Gal residues in the backbone of CX. Converting the reducing end aldehyde of the products generated by BACOVA_03433 to the alditol followed by acid hydrolysis revealed the presence of xylitol but not galactitol (Fig. 4c). These data show that BACOVA_03433 is an endo-xylanase that hydrolyses xylosidic bonds in a context found only in very complex xylans. It is possible that the enzyme targets an unusual linkage in the xylan backbone; however, this is unlikely as the enzyme was not active against β1,4-β1,3-mixed linked xylans and CX is reported to contain a backbone comprising exclusively β1,4-Xyl units^13^. To explore the specificity determinants of the GH98 enzyme further, CX was pre-treated with cocktails of side-chain cleaving hydrolases (arabinofuranosidases, xylosidases, glucuronidases and galactosidases) encoded by PUL-XylL, and the debranched forms of the xylan were then incubated with the GH98 xylanase. Only GH consortia lacking BACOVA_03417 generated a variant of CX that was hydrolysed by the GH98 enzyme (Fig. 4d). As described below BACOVA_03417 is an α-L-arabinofuranosidase that targets the O3 linked Ara*f* on backbone Xyl residues decorated at both O2 and O3 with α-L-Ara*f*. It would appear, therefore, that the double Ara*f* decoration is a component of the specificity determinant for BACOVA_03433. Wheat arabinoxylan, however, also contains this double arabinose decoration and is not a substrate for the GH98 xylanase (Fig. 4a). Thus, the specificity of BACOVA_03433 is more complex than simply Xyl units linked O2 and O3 to arabinose. Interestingly, the CX backbone contains low levels of doubly substituted residues with Xyl*p* as the O3 linked side chain (see Fig. 1 and results below). This structure is also a substrate for BACOVA_03417 (Supplementary Fig. 4a), suggesting Xyl*p* at O3 (and Ara*f* at O2) may be the critical specificity determinant of the GH98 enzyme. These Xyl*p* decorations are absent in WAX (Supplementary Fig. 4a–c), providing a possible explanation for the lack of activity of the GH98 enzyme against this substrate.

The large products generated by all three surface xylanases indicate that these enzymes recognize infrequent specificity determinants in the xylan, which, as discussed below, likely contributes to the capacity of *B. ovatus* to efficiently utilize the oligosaccharides generated by these GHs. It is particularly intriguing that the specificities of the three endo-acting enzymes are adapted to recognize the variable decorations appended to the backbone of different xylans, likely reflecting the multiple forms of the polysaccharide presented to the human large intestine.

### Surface xylan-binding proteins

PUL-XylS and PUL-XylL encode one (BACOVA_04392) and two (BACOVA_03427 and BACOVA_03429) SusD-like proteins, respectively. SusD-like proteins appear to be involved in binding and delivering oligosaccharides to their cognate SusC-like transporter for import into the periplasm^4^,^18^,^19^,^30^. Isothermal titration calorimetry showed that BACOVA_04392 bound to the relatively simple xylans WAX and BGX with moderate affinity (*K*_d_ ∼0.3–0.4 mM), but did not interact with highly decorated CX (Supplementary Table 3). The protein bound xylohexaose, but not shorter xylooligosaccharides, consistent with the long products generated by the surface GH10 xylanase encoded by PUL-XylS, BACOVA_04390 (Supplementary Fig. 3a). The SusD-like protein, derived from PUL-XylL, BACOVA_03427, bound tightly to BGX and CX (*K*_d_ ∼7–12 μM), but not WAX or linear xylooligosaccharides, although the precise target ligand for this protein is unclear (Supplementary Table 3). The other PUL-XylL SusD-like protein, BACOVA_03429, could not be expressed in a recombinant form.

In addition to the SusD-like proteins, PULs often encode surface-located glycan binding proteins (SGBPs), which are functionally analogous to SusE and F from the prototypic starch utilization system^17^,^18^,^19^,^31^. The capacity of three non-catalytic lipoproteins encoded by the xylan PULs (candidate SGBPs) to bind xylans and undecorated xylooligosaccharides was thus also explored. The data show that BACOVA_04391, from PUL-XylS, bound xylans and xylooligosaccharides with affinity increasing with chain length (Supplementary Table 3). The SGBP displayed similar specificity to the PUL-XylS SusD-like protein, albeit with higher affinity for all ligands. The crystal structure of BACOVA_04391 (PDB accession code 3ORJ) revealed a protein that contains three β-sandwich domains that are aligned in a linear orientation with respect to each other (Supplementary Fig. 5). The loops that connect the two β-sheets of the C-terminal domain form a wide cleft in which two aromatic residues, Trp241 and Tyr404, are orientated parallel to each other and are in an ideal position to make hydrophobic interactions with the two faces of a xylopyranose ring. The topology of this site centred on the parallel aromatic residues is very similar to non-catalytic CBMs that also target xylan^25^. The relative positioning of the domains in the lipoprotein BACOVA_04391 positions the ligand binding site away from the cell surface, likely to maximize efficiency of initial glycan capture.

**Figure 2:**
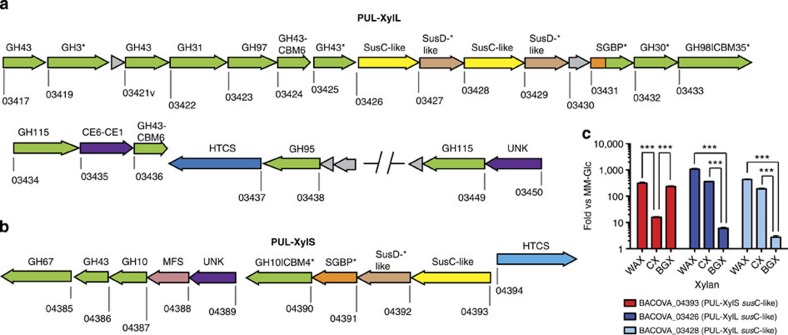
Schematic of the *B. ovatus* xylan PULs and differential expression during growth on different xylans. (**a**,**b**) schematic of PUL-XylL and PUL-XylS, respectively. Each gene is drawn to scale as a rectangle with its orientation indicated by the arrow head. The numbers below each gene is its locus tag (*bacova*_XXXXX). Genes encoding known or predicted functionalities are colour-coded and, where appropriate, are also annotated according to their CAZy family number: glycoside hydrolase (GH; green), carbohydrate esterase (CE; purple), carbohydrate-binding module (CBM). Surface located proteins are marked with an asterisk. SGBP=surface glycan binding proteins are coloured orange, or, if also in a CAZy family (BACOVA_03431; inactive GH10), are coloured half orange, half green. UNK=unknown (purple), but distant similarity to CE6 carbohydrate esterases. HTCS=hybrid two component system (light or dark blue). MFS=transporter of the major facilitator superfamily (pink). Grey=unknown function (note, there is a structure of BACOVA_03430, PDB accession code 3N91). SusC-like (yellow) and SusD-like (light tan) proteins are a defining feature of PULs and are responsible for import of complex glycans across the outer membrane^18^. SusC-like proteins are TonB-dependent transporters, while SusD-like proteins are surface lipoproteins that likely function to deliver the target glycan to their partner SusC. (**c**) Cells were grown on minimal media with polysaccharide as the sole carbon source, and levels of different *susC* transcripts (locus tags shown; used as a proxy for expression of the whole PUL) from each PUL were quantified by qRT-PCR. The *y*-axis shows the Log fold-change relative to a minimal media-glucose reference; *x*-axis labels indicate the xylans used. WAX= wheat arabinoxylan, CX=corn xylan, BGX=birchwood glucuronoxylan. CX is a highly complex xylan compared to WAX and BGX. All data were analysed by one-way ANOVA followed by Tukey’s multiple comparison test (***=*P*≤0.001).

**Figure 3:**
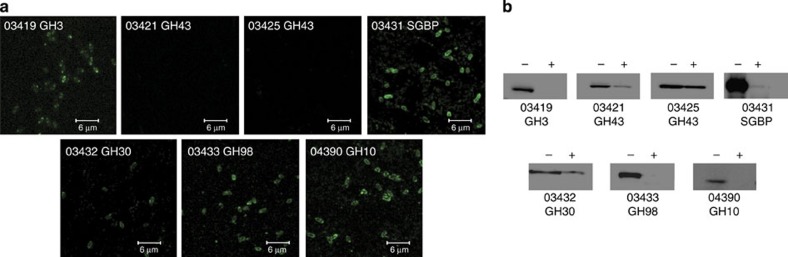
Cellular location of Xylan PUL enzymes containing a predicted N-terminal lipidation site or uncleaved transmembrane anchor. (**a**) Fluorescent microscopic images of *B. ovatus* cells cultured on WAX and incubated with polyclonal antibodies raised against recombinant BACOVA_03419 (GH3), BACOVA_03421 and BACOVA_03425 (GH43s), BACOVA_03431 (SGBP), BACOVA_03432 (GH30), BACOVA_03433 (GH98) and BACOVA_04390 (GH10). (**b**) Western blots of the *B. ovatus* cells shown in panel *a*, either untreated with Proteinase K (-) or incubated with 2 mg ml^−1^ Proteinase K for 16 h (+). The blots were probed with antibodies against the *B. ovatus* proteins indicated. The full blots are shown in Supplementary Fig. 8.

**Figure 4:**
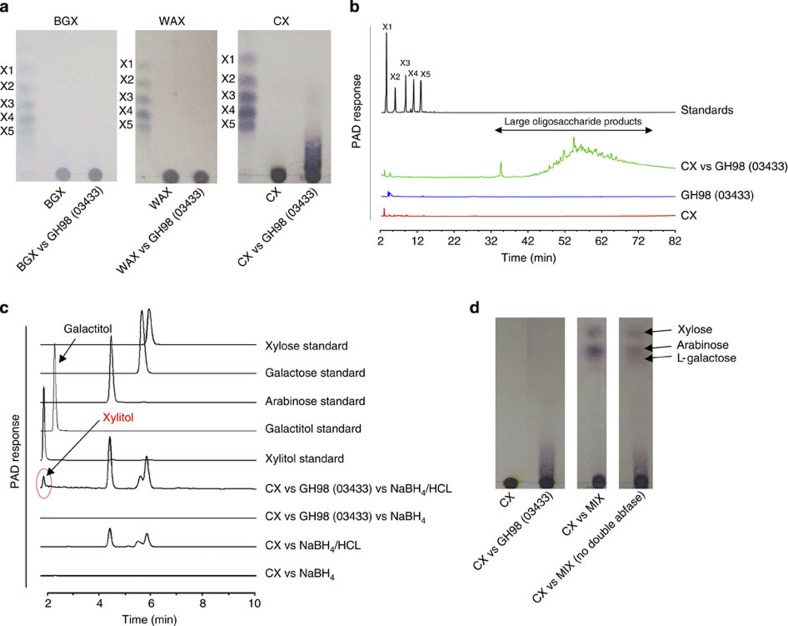
The activity of the GH98 enzyme BACOVA_03433. (**a**) Different xylans at 1% (w/v) were incubated with 1 μM of the GH98 enzyme in PBS for 16 h at 37 °C. The reaction products were subjected to thin layer chromatography and compared with xylooligosaccharide standards with a d.p. ranging from 1 to 5 (X1-X5). (**b**) HPAEC-PAD trace showing the high molecular weight oligosaccharide products of CX digestion by the GH98 endo-xylanase, separated using a sodium acetate gradient of 0-500 mM. The reaction conditions used are the same as in panel *a* Control reactions of substrate and enzyme only are shown. (**c**) CX was incubated with 5 μM of the GH98 enzyme for 24 h in 50 mM sodium phosphate buffer, pH 7.0, at 37 °C. The reaction products, and CX that was not incubated with the GH98 enzyme, were then treated with sodium borohydride, to convert the reducing end monosaccharide unit into its alditol, followed by acid hydrolysis to convert the oligosaccharides (or in the case of control CX alone) into their monosaccharide constituents. The hydrolysed products were subjected to HPAEC and compared to the migration of standard monosaccharides as well as the alditol of xylose (xylitol) and galactose (galactitol). (**d**) The GH98 enzyme was incubated with CX (lane CX vs GH98), or CX that had been pre-treated with all the side chain cleaving enzymes encoded by PUL-XylL (lane CX vs MIX), or CX that had been pre-treated with all the side chain cleaving enzymes encoded by PUL-XylL, except the GH43 enzyme BACOVA_03417 that removes O3 linked arabinose from double substituted xylose (lane CX vs MIX no double abfase).

PUL-XylL encodes two potential SGBPs, BACOVA_03430 and BACOVA_03431. BACOVA_03430 did not bind to any of the xylans or xylooligosaccharides evaluated and thus is unlikely to be a SGBP. BACOVA_03431, a surface located lipoprotein (Fig. 2), has sequence similarity to GH10 enzymes but was inactive against xylans, reflecting the loss of the catalytic nucleophile and disruption of the -2 subsite^32^. This inactive GH10 was shown to be a xylan binding protein recognizing both BGX and CX. BACOVA_03431 displayed similar specificity (but lower affinity) to BACOVA_03427, one of the SusD homologues encoded by PUL-XylL. Although no other naturally occurring ‘inactive’ GH10 members have been described, the function of BACOVA_03431 has some resonance with GH18 chitin binding lectins that lack the catalytic residues present in chitinases within this family^33^. Collectively, the data indicate that BACOVA_03431 plays a role in xylan acquisition at the cell surface.

It would appear, therefore, that the binding specificities of the SGBPs and SusD-like proteins are entirely consistent with the xylans targeted by the enzymes encoded by the two PULs. Thus, the PUL-XylS SusD-like protein and SGBP target GXs and long chain undecorated xylooligosaccharides, whereas the equivalent PUL-XylL proteins recognize the more complex structures found in cereal xylans such as CX.

### Enzymatic debranching of xylans in the periplasm of *B. ovatus*

The xylan-derived xylooligosaccharides entering the periplasm are degraded by a suite of primarily exo-acting enzymes encoded by PUL-XylS and PUL-XylL, which are described below.

BACOVA_03417 GH43 generated arabinose from AXs, CX and sugar beet arabinan, before and after treatment with an arabinofuranosidase that removes only single Ara*f* decorations (*Cj*Abf51A^34^; Abfase-S), (Supplementary Fig. 4a,b and Supplementary Table 4), while the enzyme displayed no activity against WAX pre-treated with Abfase-D (*Hi*AXHd3^35^), a fungal enzyme that targets the O3 arabinose of Xyl units decorated at both O2 and O3 with Ara*f* (Supplementary Fig. 4c). BACOVA_03417 shows ∼30% sequence identity with *Hi*AXHd3, with all the residues that contribute to specificity in the active site and the O2 Ara*f* specificity pocket conserved in the two enzymes. It is likely, therefore, that the *B. ovatus* enzyme targets the O3 linkage in the double substitutions, using the O2 Ara*f* as a critical specificity determinant as occurs in *Hi*AXHd3^35^.

Incubation of BACOVA_03421 and BACOVA_03425 GH43s with WAX or CX released arabinose (Supplementary Fig. 4d,e and Supplementary Table 4), while neither enzyme was active against WAX pre-treated with the mono-specific GH51 arabinofuranosidase *Cj*Abf51A^34^ (Supplementary Fig. 4f). These data show that both BACOVA_03421 and BACOVA_03425 are arabinofuranosidases that target single substitutions (either O2 or O3 linked) on the xylan backbone.

It should be noted that the three GH43 arabinofuranosidases (BACOVA_03417, BACOVA_03424 and BACOVA_03425) also released a small amount of xylose from CX but not from WAX (Supplementary Fig. 4), reflecting β1,3-Xyl side chains in CX (as single decorations and as a component of an O2 Ara*f* and O3 Xyl*p* double decoration, Fig. 1). These data are consistent with the observation that the active sites of GH43 enzymes bind Xyl*p* and Ara*f*^35^.

PUL-XylL encoded two other GH43 enzymes, BACOVA***_***03424 and BACOVA_03436, that contained a C-terminal family 6 CBM^16^,^36^ and displayed ∼46% sequence identity with each other. Both enzymes displayed limited activity against CX only, generating very low concentrations of a short oligosaccharide. The functional significance of these two enzymes is unclear.

BACOVA_03449 is a GH115 α-glucuronidase from PUL-XylL that was previously shown to cleave internally substituted glucuronic and methyl-glucuronic acid from GXs, GAXs and glucurono-xylooligosaccharides^37^. These data are consistent with the predicted periplasmic location of BACOVA_03449. This supports the view that the target substrates for the GH115 α-glucuronidase are internally substituted oligosaccharide products generated by the surface-located GH30 or GH98 endo-xylanases, which are also from PUL-XylL. BACOVA_04385 from PUL-XylS, is a GH67 α-glucuronidase and, like other members of this family, is active only on terminally substituted xylo-oligosaccharides (Supplementary Table 5)^38^. This is consistent with the products generated by the surface GH10 endo-xylanase, BACOVA_04390, which is also encoded by PUL-XylS.

### Novel xylan debranching enzymes

**Figure 5:**
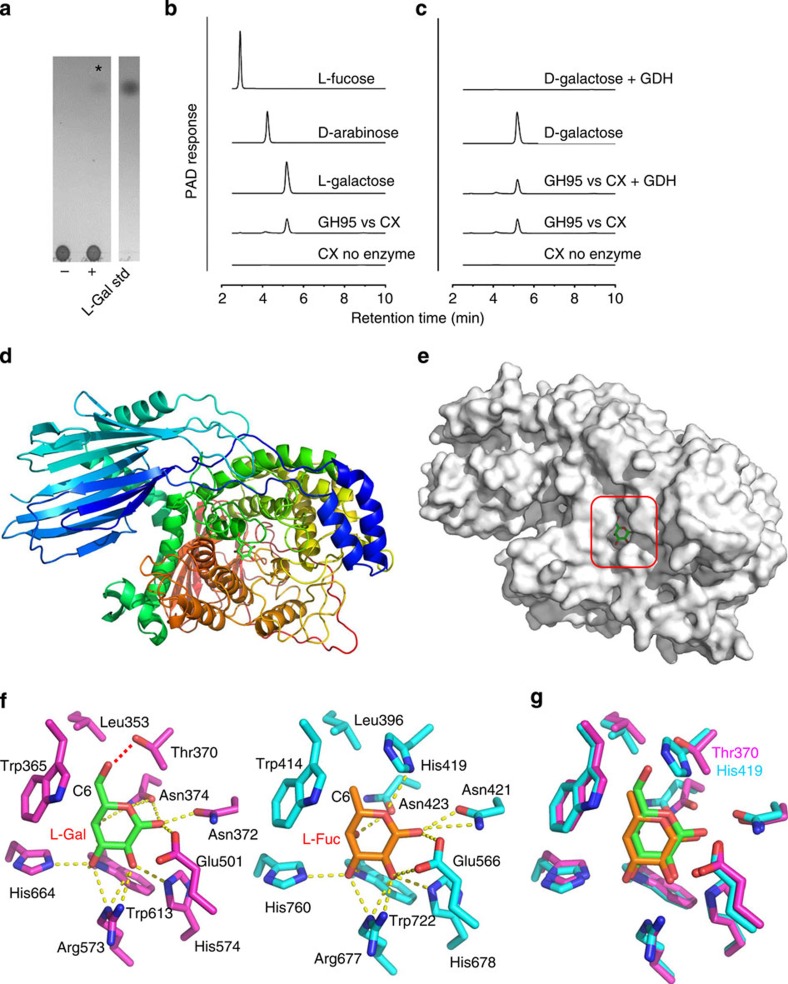
Activity and structure of BACOVA_03438 GH95 α-L-galactosidase and comparison to the *Bifidobacterium bifidum* GH95 α-L-fucosidase. (**a**,**b**) TLC and HPAEC profiles, respectively, of the reaction product (L-galactose) generated by incubating CX at 1 mg ml^−1^ with 1 μM BACOVA_03438 for 16 h in 20 mM sodium phosphate buffer, pH 7.0, at 37 °C. In (**a**) the lanes are as follows: (−) CX no enzyme; (+) CX incubated with BACOVA_03438. The asterisk marks the position of the single reaction product released from CX by the GH95. (**c**) The product of the GH95 is resistant to oxidation by D-galactose dehydrogenase (GDH). (**d**) Schematic of BACOVA_03438, colour-ramped from blue (N-terminus) to red (C-terminus), with L-Gal bound in the active site (carbohydrate carbons shown as green sticks). (**e**) Solvent exposed surface representation of *B. ovatus* GH95. The active site pocket that houses β-L-Gal product is boxed. (**f**) Side chains of the active site residues of BACOVA_03438 (magenta carbons) and the GH95 α-L-fucosidase (blue carbons; PDB: 2EAE) from *Bifidobacterium bifidum*, bound to L-Gal (green carbons) and L-Fuc (orange carbons), respectively. Predicted hydrogen bonds between the amino-acid side chains and carbohydrates are shown as yellow dotted lines, except for the H-bond between the Oγ of BACOVA_03438 Thr370 and O6 of L-Gal, which is shown as a red dotted line. (**g**) Overlay of the active site residues displayed in panel (**f**) with the side-chains of the Thr/His polymorphism that may play a role in specificity for L-Gal over L-Fuc labelled. A stereo image of a portion of the electron density map is shown in Supplementary Fig. 9.

As discussed above the two xylan PULs encode debranching enzymes located in GH families typically associated with xylan degradation (GH43, GH67 and GH115) that cleave α-L-arabinofuranosyl and α-D-glucuronyl linkages. In addition, PUL-XylL encodes three periplasmic proteins that belong to GH31, GH97 and GH95, families that have not previously been implicated in the deconstruction of the polysaccharide. Assays showed that the GH31 enzyme, BACOVA_03422, released xylose from CX but displayed no activity against a range of other xylans (Supplementary Fig. 6). As GH31 enzymes target α-D-glycosides^39^, BACOVA_03422 is likely an α-xylosidase, consistent with the presence of α-D-Xyl in CX^13^. BACOVA_03422 is ∼3,000-fold more active against CX than a xyloglucan oligosaccharide (Supplementary Table 6), demonstrating that the enzyme is a xylan-specific α-xylosidase. In contrast, a previously characterized GH31 α-xylosidase, *Cj*Xyl31A^40^, exhibited minimal activity against CX, strongly preferring xyloglucan oligosaccharide (Supplementary Table 6). These data show that the xylan specificity displayed by the BACOVA_03422 enzyme is not a general property of GH31 enzymes. The GH97 enzyme, BACOVA_03423, was active against 4-nitrophenyl-α-D-galactopyranoside, consistent with the presence of α-D-Gal in some highly complex xylans^13^. The GH95 enzyme BACOVA_03438, although located in an ‘α-L-fucosidase specific family’^41^, released exclusively L-Gal from CX, displaying only trace activity against oligosaccharides containing terminal α-L-Fuc residues (Fig. 5a–c and Supplementary Table 7). Conversely, a previously characterized GH95 α-1,2-L-fucosidase (AfcA)^42^ exhibited minimal α-L-galactosidase activity against CX (Supplementary Table 7), showing that GH95 enzymes can be either L-Gal or L-Fuc specific. Although these data provide an initial example of a glycosidase specific for L-galactose, it should be noted that the GH117 family contains enzymes that target the 3,6-anhydro form of the sugar^43^.

### Crystal structure of the GH95 α-L-galactosidase

The crystal structure of the α-L-galactosidase, BACOVA_03438, displays a central (α/α)_6_-helical barrel domain flanked by β-sandwich domains at the N- and C-termini, respectively. In the structure of the protein-product complex, β-L-Gal (GH95s are inverting enzymes), which adopts a ^*1*^*C*_*4*_ geometry typical of L-monosaccharides, is buried in the central cavity of the helical barrel domain, (Fig. 5d–g). Structural analyses in combination with mutational experiments revealed that the highly conserved Glu501 acts as the general acid catalyst in a single displacement (inverting) mechanism (Fig. 5f and Supplementary Table 7). No carboxylic acid residue was found at the appropriate position for a general base catalyst, similar to the *Bifidobacterium bifidum* GH95 α-L-fucosidase, AfcA. It was proposed that in AfcA two asparagine residues activate a water molecule that is suitably located to perform a nucleophilic attack on the C1 atom of L-fucose in the active site^42^, which is in sharp contrast with typical GHs where Asp or Glu act as the catalytic base^44^. The proposed catalytic asparagines in AfcA (Asn421 and Asn423) are structurally conserved in the GH95 α-L-galactosidase (Asn372 and Asn374), suggesting a similar role in BACOVA_03438 (Fig. 5f,g). Almost all of the residues that interact with the pyranose ring of L-Gal in BACOVA_03438 are conserved in the AfcA fucosidase, although, intriguingly, Thr370 in the L-galactosidase is replaced with His419 in the fucosidase (Fig. 5f,g). In AfcA His419 contributes to the hydrophobic sheath in which the C6 methyl group of the Fuc is housed, while in BACOVA_03438 the γO of Thr370 makes a direct polar contact with the O6 of L-Gal, the only part of the monosaccharide that differs from L-Fuc. The importance of the Thr370 in BACOVA_03438 is demonstrated by the complete loss of activity of the T370H mutant against CX (Supplementary Table 7). While the Thr/His polymorphism may be a specificity determinant for galactosidase and fucosidase activity within GH95, the paucity of enzymes characterized in this family prevents a definitive conclusion regarding its functional significance. Indeed, the observation that the T370H mutation does not increase fucosidase activity suggests that L-Gal to L-Fuc specificity is driven by more than just the −1 site (Supplementary Table 7). Significantly, the leaving group subsite (+1 subsite) is not conserved in the two enzymes (inferred in the case of BACOVA_03438), and it is possible that substrate binding in this region contributes to enzyme specificity. A key role for the +1 subsite in the two enzymes is consistent with their lack of activity against 4-nitrophenyl-α-L-fucopyranoside (AfcA) and 4-nitrophenyl-α-L-galactopyranoside (BACOVA_03438).

### Hydrolysis of undecorated xylooligosaccharides

Breakdown of debranched xylooligosaccharides, derived through the action of the exo-acting enzymes described above, is mediated by enzymes encoded by PUL-XylS. A typical GH10 endo-acting xylanase (BACOVA_04387) rapidly generates oligosaccharides in the periplasm with a d.p. of primarily 1 to 4 (Supplementary Fig. 3b and Supplementary Table 2). These small xylooligosaccharides are substrates for the cytoplasmic GH43 β-xylosidase, BACOVA_04386 (Supplementary Table 1 and Supplementary Fig. 2c). The spatial separation of the β-xylosidase from the enzymes that generate its substrate may reflect a selection advantage as less energy would be required to transport a small number of xylooligosaccharides across the inner membrane (likely via the PUL-encoded MFS class transporter, BACOVA_04388, Fig. 2b), compared with a larger number of xylose units if the β-xylosidase was periplasmic. It is also possible that the different cellular locations of the xylosidase and xylanase maximizes the signal concentration for the PUL-XylS HTCS sensor-regulator (BACOVA_04394), which recognizes short xylooligosaccharides^4^.

### The function of the two xylan PULs in *B. ovatus*

**Figure 6:**
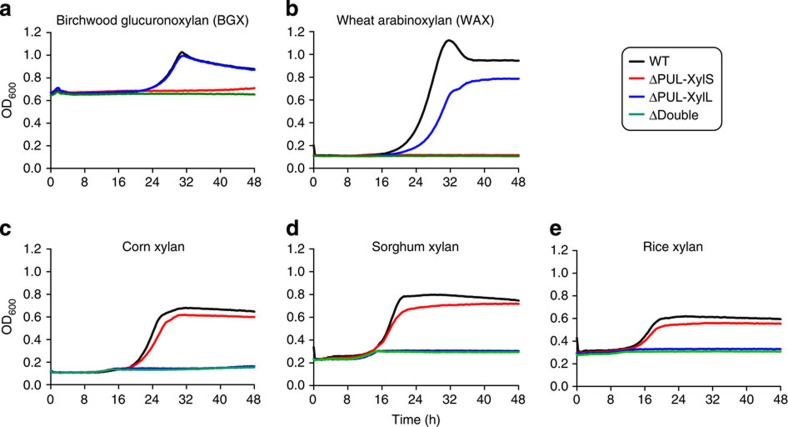
Growth profile of wild type and mutants of *B. ovatus* on xylans. WT and PUL deletion mutants were cultured in minimal media containing different xylans at 0.5% (w/v) as the sole carbon source. Birchwood glucuronoxylan (BGX; (**a**)) and wheat arabinoxylan (WAX; (**b**)) are relatively simple, sparsely decorated structures, while corn (**c**), sorghum (**d**) and rice (**e**) xylans are more complex heavily decorated glucuronoarabinoxylans (GAXs). The high starting OD for BGX is due to the background turbidity of the polysaccharide. A total of 6 replicate cultures were monitored at 20 min intervals for each substrate and used to generate the average growth curves shown.

The role of PUL-XylS and PUL-XylL in xylan degradation is illustrated by the growth profiles of mutant strains. Deletion of PUL-XylS (ΔPUL-XylS) did not influence the growth of *B. ovatus* on complex xylans (GAXs) from corn, rice and sorghum, but prevented growth on simpler xylans (BGX or WAX) (Fig. 6). By contrast, inactivation of PUL-XylL (ΔPUL-XylL) prevented growth on the three complex GAXs but did not significantly affect the utilization of simpler BGX or WAX (Fig. 6). The somewhat higher final density observed for the wild-type bacterium on WAX vs ΔPUL-XylL is likely due to the inability of this mutant to remove the arabinose decorations from this substrate. The growth profile of the mutants are consistent with the biochemical data presented above showing that PUL-XylL encodes the only endo-acting enzyme (GH98) able to cleave the backbone of CX (and likely other complex GAXs), a prerequisite for the utilization of these polysaccharides (see below). Similarly, the inability of the PUL-XylS mutant to utilize simple xylans such as GXs and AXs, where the xylan backbone is the major component, is consistent with the locus encoding the key endo-acting surface GH10 (BACOVA_04390) that hydrolyses the backbone of sparsely decorated xylans. It is somewhat surprising, however, that ΔPUL-XylS is able to efficiently utilize GAX even though the mutant is unable to fully depolymerize the xylan backbone. This likely reflects the extensive side chains present in CX and other complex GAXs, where the xylose backbone constitutes only a relatively minor component of the total monosaccharide units in these polysaccharides.

**Figure 7:**
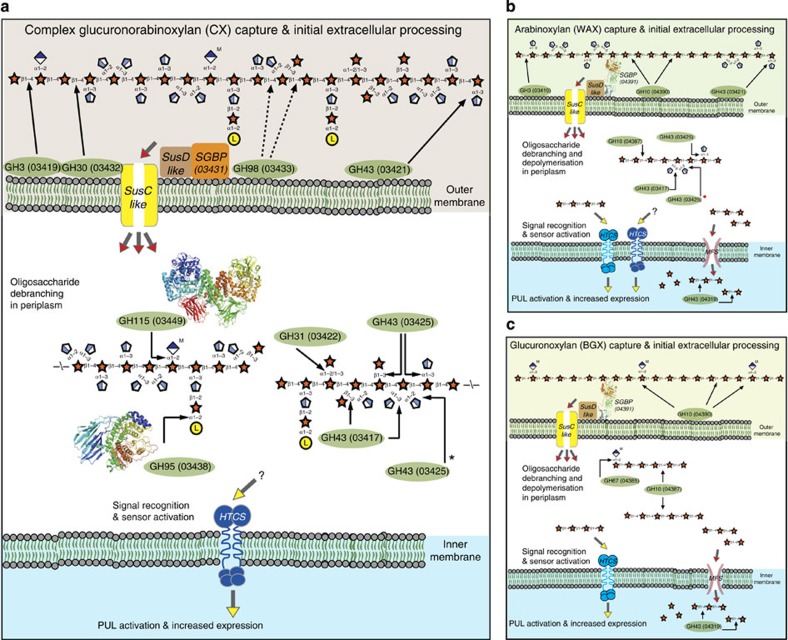
Models for the degradation of different forms of xylan by *B. ovatus*. In the upper part of each panel the monosaccharides and linkages in the main classes of xylan are shown and are represented in their Consortium for Functional Glycomics format^69^. The lower parts of each panel show the model for degradation of complex GAXs like CX (**a**) AXs like WAX (**b**) and GXs like BGX (**c**) The red asterisk next to BACOVA_03425 GH43 indicates that the single specific arabinofuranosidase acts after BACOVA_03417 (α1,3 double-specific arabinofuranosidase) to remove the remaining α1,2 linked L-arabinose. The black arrows indicate examples of the linkages cleaved by the enzymes, shown in green ovals and identified by their glycoside hydrolase family and locus tag. In panel **a**, the dotted arrows from the GH98 enzyme identify possibly linkages hydrolysed by the xylanase. Although not shown on the CX model, the PUL-XylS apparatus will be present, albeit at low levels (see Fig. 2c). A schematic of the crystal structures, colour-ramped from blue (N-terminus) to red (C-terminus), of two of the xylan degrading enzymes, determined in this study (BACOVA_03438 GH95; PDB accession code 4UFC) and in a recent report^37^ (BACOVA_03449 GH115; PDB accession code 4C91) are shown. The red arrow heads indicate glycan transport between cellular locations, while the yellow arrow heads show the ligand (or potential ligand in case of the question mark) that binds and activates the hybrid two component system (HTCS) encoded by PUL-XylS (BACOVA_04394, light blue HTCS; activating ligand xylotetraose^4^) or PUL-XylL (BACOVA_03437, dark blue HTCS; activating ligand likely an arabino-xylooligosaccharide). MFS is a member of the Major Facilitator Superfamily of transport proteins, and SGBP signifies the two surface xylan binding proteins encoded by the xylan PULs, BACOVA_03431 (inactive GH10, PUL-XylL) and BACOVA_04391 (PUL-XylS). A schematic of the crystal structure of BACOVA_04391 (PDB accession code 3ORJ), colour-ramped from blue (N-terminus) to red (C-terminus), is shown. Note only one of the two SusC/D pairs from PUL-XylL is shown for clarity.

To summarize, using the expression, localization and biochemical data presented above Fig. 7 shows the proposed models for the acquisition and breakdown by *B. ovatus* of the different classes of xylan presented to the colonic microbiota.

### The conservation of PUL-XylS and PUL-XylL in Bacteroidetes

**Figure 8:**
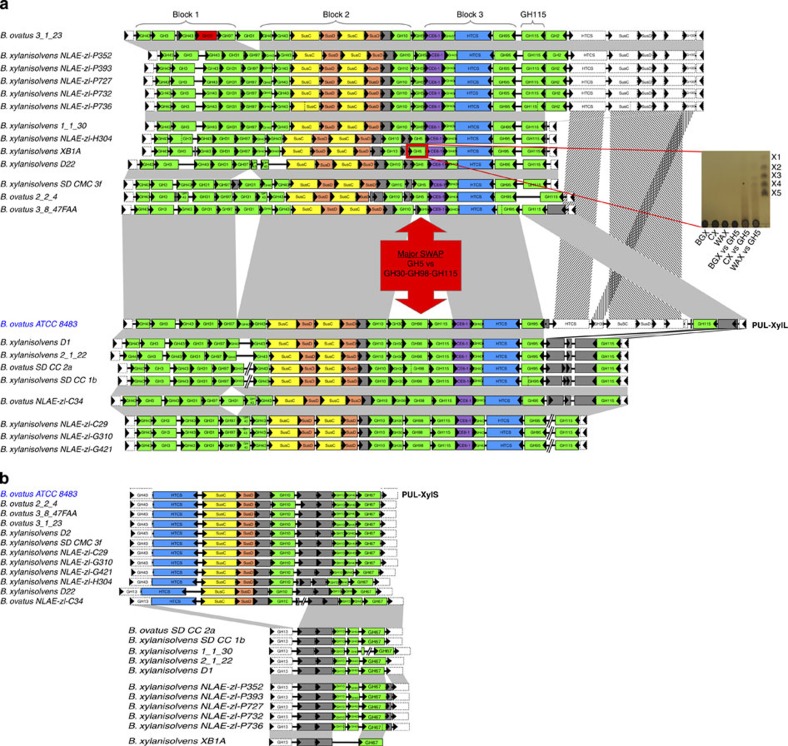
Conservation of the *B. ovatus* ATCC 8483 xylan PULs in other *Bacteroides* species. CAZymes (green, CBM modules were omitted for clarity), *sus*C-like (yellow), *sus*D-like (orange) and the HTCS sensor-regulator (blue) genes are colour-coded. Genes encoding proteins of unknown function or non-CAZymes (for example, surface glycan binding protein and MFS carbohydrate importer in PUL-XylS) are shown in grey. (**a**) PUL-XylL. Vertical dotted lines indicate split gene models, while missing gene models are shown in red. Microsyntenic segments are depicted by grey connectors, and include adjacent conserved non-PUL members (shown with dotted lines). Eighteen genes are conserved across all strains, and 17 of these form three highly conserved blocks indicated on top. The major difference between the two groups of the GH98 for GH5_21 swap is indicated. Inset shows a TLC of the activity of the recombinant *B. xylanisolvens* XBA1 GH5_21 enzyme (BXY_29320; boxed in red) against different xylans (1 μM final enzyme vs 0.5% xylan in 20 mM sodium phosphate buffer, pH 7.0, 16 h at 37 °C). The *B. xylanisolvens* GH5 enzyme displays endo-xylanase activity against CX, producing a smear of large oligosaccharide products similar to the product profile observed for the *B. ovatus* GH98 enzyme, BACOVA_03438. The GH5 xylanase also hydrolyses WAX, unlike the GH98 enzyme, but displays no activity against BGX. X1-X5 indicates xylooligosaccharide standards. (**b**) PUL-XylS. The lower part of the panel shows the strains that contain only the second half of PUL-XylS that encodes the intracellular (periplasmic and cytoplasmic) enzymes only.

To identify PULs similar to PUL-XylL and -XylS in other genomes, we examined not only the gene family composition but also gene synteny in 260 Bacteroidetes species (Supplementary Table 8). Comparative genomics revealed a high degree of conservation of PUL-XylL in *B. ovatus* and *B. xylanisolvens* strains. Indeed, PULs with a high level of synteny with PUL-XylL was observed in 22 of the 23 strains examined. In these PULs, 17 genes show strict conservation with those present in PUL-XylL, which are arranged into three syntenic blocks (Fig. 8a). These 22 PUL-XylL homologues can be subdivided into two groups that differ by the presence of a tandem array of three genes. The three genes in group one PUL-XylLs, which encode GH30_8 (subfamily 8)^45^, GH98 and GH115 enzymes (including *B. ovatus* ATCC 8483), are replaced in group two PUL-XylLs by a single gene encoding a GH5 subfamily 21 (GH5_21)^46^ glycanase (Fig. 8a). Significantly, the GH5_21 enzyme from *B. xylanisolvens* XBA1 displays activity on CX (Fig. 8a inset), similar to the *B. ovatus* GH98 enzyme BACOVA_03433. This suggests that both forms of PUL-XylL retain the capacity to hydrolyse the backbone of complex GAXs without the need for extensive debranching. The high synteny described above for PUL-XylL in the *B. ovatus* and *B. xylanisolvens* strains was not found in any of the other 260 Bacteroidetes genomes analysed. In particular, the distribution of the GH98-encoding gene is extremely narrow, being present in only five of the 260 genomes analysed.

With respect to PUL-XylS there is perfect conservation of the synteny of the genes encoding the GH67 α-glucuronidase, GH43 β-xylosidase and periplasmic GH10 xylanase in all 23 *B. ovatus* and *B. xylanisolvens* strains (Fig. 8b). In 11 of these strains, however, the genes encoding the surface GH10 xylanase, SusD-like, SusC-like and HTCS proteins were absent. The loss of these critical components in these strains is likely to render the PULs non-functional. No other candidate PUL with complete conservation of all PUL-XylS components could be found in the other Bacteroidetes that we examined. The closest candidate PULs were found in *B. intestinalis*, *B. gallinarum*, *B. oleiciplenus* and *Proteiniphilum acetatigenes*. Interestingly, some of these PULs are adjacent to gene families that are found in PUL-XylL that encode GH5_21, GH10, GH31, GH43, GH95 or GH115 enzymes (the degree of synteny is uncertain due to limited scaffold assemblies), suggesting a possible common evolutionary origin for the *B. ovatus* XylL- and XylS- PULs. Overall, of the 23 *B. ovatus* and *B. xylanisolvens* strains, only five have both XylL- and XylS-PULs, illustrating the dynamic acquisition (and possibly loss and/or reshuffling) of PULs by Bacteroidetes.

### Loss of xylan degradation products from the cell surface

**Figure 9:**
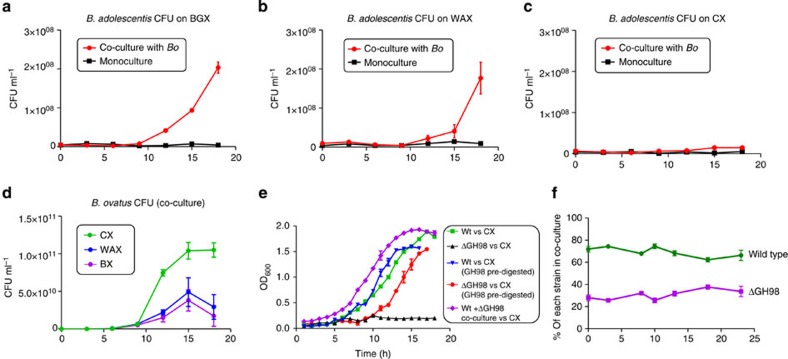
The ability of other members of the microbiota to use PBPs released by *B. ovatus* during growth on xylan is determined by the complexity of the polysaccharide. *Bacteroides ovatus (Bo)* and *Bifidobacterium adolescentis* were co-cultured on BGX (**a**) WAX (**b**) and CX (**c**) and the number of CFUs of each determined at different points on the growth curve. (**d**) Shows the CFU ml^-1^ of *B. ovatus* alone at different phases of growth. Note, when the *Bifidobacterium* alone was grown on digested xylans (see Supplementary Fig. 8), the CFU ml^−1^ at late exponential phase (OD_600_ ∼1.2) was ∼8.0 × 10^8^, indicating that ∼25% of the total xylan is used by *B. adolescentis*. (**e**) Growth of *B. ovatus* wild type and the ΔGH98 mutant (BACOVA_03433) on CX (0.5% w/v). Pre-digested indicates the CX was digested to completion with the GH98 xylanase prior to addition to the media. (**f**) Tagged strains of wild-type *B. ovatus* and the ΔGH98 mutant were co-cultured on CX (see panel *e* for growth curve). Samples were taken at different time points and qPCR with primers unique to each strain was used to determine the ratio of each in the culture. Each data point is the average and s.d. of triplicate growths.

Similar to other characterized *Bacteroides* polysaccharide degrading systems, the xylan degrading apparatus of *B. ovatus* appears to be optimized to maximize intracellular breakdown, which may indicate that the bacterium adopts a ‘selfish’ strategy when deconstructing the hemicellulose^17^,^18^,^19^. In contrast with this selfish hypothesis is the observation that on simple xylans, such as BGX and WAX, *B. ovatus* was able to support the growth of *Bifidobacterium adolescentis* strain ATCC 15703, which utilizes simple linear and arabino-xylooligosaccharides but not xylans^47^ (Fig. 9a,b and Supplementary Fig. 7). The *Bacteroides*, however, was unable to promote the growth of the *Bifidobacterium* on highly complex xylans such as CX (Fig. 9c). It could be argued that this differential capacity of *B. ovatus* to support the growth of *B. adolescentis* reflects the extent to which oligosaccharides from complex (CX) and simple (BGX and WAX) xylans are released into the culture medium and are thus available to the *Bifidobacterium*. It should be emphasized, however, that as we show here, CX breakdown requires a suite of enzymes not associated with canonical xylan degrading systems. Indeed only a limited number of closely related species of *Bacteroides* appear to contain these additional enzymes (see above and Fig. 8a). Thus, the inability of *B. ovatus* to support growth of *B. adolescentis* on CX may simply reflect the fact that the *Bifidobacterium* lacks the necessary apparatus to utilize this complex xylan. To explore this possibility a BACOVA_03433 GH98 mutant was constructed in which the key catalytic residues of the GH98 enzyme (Glu361 and Asp467) had been replaced with alanine. The mutant (ΔGH98) lacked an active surface GH98 xylanase and was unable to cleave the backbone of CX, preventing growth on the complex GAX (Fig. 9e). The *B. ovatus* variant, however, retained the other components of the xylan degrading apparatus, and was thus able to grow well on CX that had been pre-digested with recombinant GH98 (Fig. 9e). Co-culture of ΔGH98 with wild-type *B. ovatus* on intact CX supported the growth of both strains, demonstrating that PBPs are indeed released into the culture medium by wild-type *B. ovatus* during growth on CX (Fig. 9f). Thus, the inability of *B. adolescentis* to grow on CX in the presence of *B. ovatus* does not reflect the release of an inadequate supply of xylan-derived PBPs, but indicates that the *Bifidobacterium* lacks the apparatus required to utilize these highly complex oligosaccharides. This is consistent with the finding that *B. adolescentis* is unable to grow on CX that had been pre-digested with either the GH98 alone or all the surface located enzymes encoded by PUL-XylL (Supplementary Fig. 7).

Currently the degree to which glycan degradation is mediated by synergistic interactions between different members of the gut microbiota is unclear. It has been proposed that there are keystone organisms belonging to the Firmicute phylum that make recalcitrant glycans, such as resistant starch, available to other members of the ecosystem^48^. Similarly, a recent study showed that the products released during degradation of highly accessible glycans (branched starch and fructans) by *Bacteroides* spp. were shared with recipients that alone are unable to utilize these polysaccharides^49^. In contrast, *B. thetaiotaomicron* utilizes complex yeast mannans in a selfish manner as breakdown occurs almost entirely intracellularly, with no evidence of sharing of PBPs with other *Bacteroides* spp.^27^. This study extends our understanding of resource allocation in the microbiota by showing that selfish glycan utilization is not dependent solely on an ability to retain PBPs within the cell, but can be determined by the complexity of the enzyme systems required to access the extracellular oligosaccharides generated.

## Discussion

This report provides a model for the microbial utilization of xylan, a key polysaccharide present in the human diet. The data demonstrate that current understanding of xylan degradation, based primarily on the breakdown of relatively simple xylans by soil microbial ecosystems, is inadequate when considering the complex GAXs found in the human diet^22^. It is evident that *B. ovatus* has acquired an extensive range of enzymes, several from GH families that had not previously been implicated in xylan breakdown, to assemble an efficient catalytic apparatus capable of deconstructing highly complex forms of the polysaccharide. It is significant that the genetic basis for xylan degradation is organized into two distinct loci regulated by different signals. This provides *B. ovatus* with a flexible strategy to degrade the hemicellulose. When presented with a simple linear xylan or GX a minimum enzyme cocktail is assembled (that is, PUL-XylS), while PUL-XylL is activated only in response to complex GAXs (Fig. 2c). It is likely, therefore, that the distribution of these two PULs within human microbiomes provides insight into the nature of the diet consumed by the respective host.

Using xylans that contain variable decorations but a conserved core backbone, we have shown that it is the complexity of the target polysaccharide that determines whether other members of the HGM can utilize the PBPs released by *B. ovatus* during growth on different xylans. Thus, the relatively simple oligosaccharides that are released during growth on WAX and BGX can be used by *B. adolescentis*, while the more complex molecules released from CX cannot be accessed by the *Bifidobacterium*. Indeed, our analysis of Bacteroidetes genomes suggests that very few organisms, outwith *B. ovatus* and *B. xylanisolvens*, have a sufficiently complex xylan degrading system to utilize the PBPs released from CX by these two *Bacteroides* species. It is possible that these complex glycans are only used in a nutritional crisis when competition for resources is intense within the microbiota. Recently, it has been shown that *Bacteroides* *thetaiotaomicron* displays a degradative hierarchy in which some simple glycans are prioritized above more complex polysaccharides^50^. Understanding the mechanisms by which different glycans are degraded by the microbiota, specifically whether they are public-good products or are used exclusively by the degrading organism, is critical to developing nutraceutical strategies to manipulate the structure of the microbiota to maximize human health. For example, complex GAXs will favour the growth of *B. ovatus,* which produces propionate, a molecule that reduces lipogenesis in the host^51^. In contrast, the degradation of simple xylans by *B. ovatus* will also promote the growth of *Bifidobacteria*, which can lead to the production of butyrate, a molecule that is known to maintain the health of the intestinal epithelium^52^. Thus, it may be possible to develop bespoke xylan-based diets that are designed to have different beneficial effects on human health.

## Methods

### Isolation of corn xylan

Oven-dried corn fibre sample was kindly provided by ADM Research (North America), which was further oven-dried and ground to a 20-mesh particle size using a Wiley mill^53^. The ground corn fibre sample was extracted with hexane to remove oil and treated with thermostable α-amylase (a gift from Novo Nordisk Bioindustrials, Danbury, CT) at 90-95 °C to hydrolyse the starch present in the fibre^54^,^55^. CX was isolated from de-oiled and de-starched corn fibre following the alkaline hydrogen peroxide technology of Yadav *et al.*^56^ with the following modifications. De-oiled and de-starched corn fibre (50 g) was mechanically stirred into water (1.0 l) and NaOH (8 ml from 50% solution), Ca(OH_2_) (3.8 g) and 42 ml of 30% H_2_O_2_ were carefully added in an open beaker in a fume hood. The mixture was boiled with mechanical stirring for 1 h. During the reaction, its pH was kept at 11.5 by addition of 50% NaOH as needed. After cooling the hot reaction mixture by stirring at room temp for an additional half an hour, it was centrifuged at 6,000 *g* for 20 min and the supernatant was separated from the residue by decantation. The pH of the alkaline H_2_O_2_ extract was then adjusted to 4.0–4.5 by adding conc. HCl to precipitate Hemicellulose A (acid-insoluble arabinoxylan, ‘Hemi. A’), which was collected by centrifugation at 10,000 *g* for 30 min and discarded. Two volumes of ethanol (2.0 l) were gradually added to the supernatant (1.0 l), obtained after Hemi. A removal, with stirring to precipitate the major arabinoxylan fraction (CX). The CX was allowed to settle out as a white flocculent precipitate at the bottom of the beaker for 10–15 min. The clear alcohol/water mixture above the precipitate was removed by decantation. The white flocculent precipitate was collected by filtration and dried in a vacuum oven at 50 °C overnight.

The other xylans used in this study were low viscosity wheat arabinoxylan (WAX, Megazyme International) and birchwood glucuronoxylan (BGX; Sigma Aldrich).

### Cloning, expression and purification of recombinant proteins

The open-reading frames encoding mature forms of the proteins used in the study were amplified from *Bacteroides ovatus* ATCC 8483 genomic DNA by PCR using appropriate primers (Supplementary Table 9), which introduce BamHI/HindIII and NdeI/XhoI sites to the flanks of the target genes. The amplified DNA was cloned into *Escherichia coli* expression vectors (see Supplementary Table 9) such that the encoded recombinant proteins contain either an N- or C-terminal His_6_-tag. Site-directed mutants were made using the Quikchange kit (Agilent), using primers shown in Supplementary Table 10. The recombinant proteins were produced in *E. coli* BL21 (DE3) or Tuner (Novagen) cells cultured in LB broth containing ampicillin (100 μg ml^−1^) or kanamycin (50 μg ml^−1^) at 37 °C. Cells were grown to mid-exponential phase (OD_600_ ∼0.6), at which point isopropyl β-D-thiogalactopyranoside was added to a final concentration of 1 mM (BL21) or 0.2 mM (Tuner), and the cultures were incubated for a further 16 h at 16 °C. The cells were harvested by centrifugation, sonicated and His_6_-tagged recombinant protein were purified from cell-free extracts by immobilized metal ion affinity chromatography using Talon resin (Clontech) and standard methodology as described previously^37^. For crystallographic studies BACOVA_03438 was further purified by size exclusion chromatography using a Superdex 75 column (GE Healthcare Life Sciences). All proteins were purified to electrophoretic homogeneity as judged by SDS–PAGE and their concentrations determined from their calculated molar extinction coefficient at 280 nm using a NanoDrop 2000c (Thermo Scientific).

### Growth of anaerobic bacteria

*Bacteroides ovatus* ATCC 8483 was routinely grown in tryptone-yeast extract-glucose (TYG) medium or on brain-heart infusion (BHI; Beckton Dickinson) agar plus porcine hematin (1.2 μg ml^−1^; Sigma-Aldrich)^4^,^57^. *Bifidobacterium adolescentis* ATCC 15703 was grown on clostridial nutrient medium (CNM; Sigma-Aldrich) plus hematin. Antibiotics were added as appropriate: erythromycin (25 μg ml^−1^) and gentamicin (200 μg ml^−1^). *Bacteroides* minimal medium (MM) was formulated as described previously^3^ as was *Bifidobacterium* MM^58^. Cells were grown in an anaerobic cabinet (Whitley A35 Workstation; Don Whitley, UK). Cultures (5 ml) were routinely grown in glass test tubes and the OD_600_ monitored using a Biochrom WPA cell density meter. Growth of *B. ovatus* wild type and PUL-knockout mutants was measured on various xylan preparations by mixing the autoclave-sterilised polysaccharides (0.5% final concentration) with minimal medium and monitoring growth continuously as previously described^4^.

### Quantitative RT-PCR (qPCR)

Comparison of the levels of *sus*C transcript expression from each of the xylan PULs was performed by qPCR. Previous studies have shown the *sus*C genes are a good proxy for expression of their cognate PUL^4^. *B. ovatus* was cultured in 5 ml of minimal media containing 0.5% (w/v) carbon source, as described above. Triplicate bacterial cultures were harvested at mid-log phase (OD_600_ ∼0.8) and placed in RNAprotect (Qiagen), then stored at −80 °C overnight, before purification with RNeasy kit (Qiagen). RNA purity was assessed spectrophotometrically, and 1 μg of RNA was used immediately for reverse transcription (QuantiTect Reverse Transcription kit, Qiagen). Quantitative RT-PCR was performed in a 96-well plate on a LightCycler 480 System (Roche) with FastStart Essential DNA Green Master (Roche) using the primers shown in Supplementary Table 11. Reactions were carried out in 10 μl, consisting of 5 μl SYBR Green mix, 20 ng of cDNA, and 1 μM (*sus*C genes) or 0.125 μM (16 S rRNA) primer mix. Reaction conditions were 95 °C 600 s, followed by 45 cycles of 95 °C for 10 s, 55 °C for 10 s, 72 °C for 10 s. Cq values were calculated using LightCycler 480 SW 1.5. Data were normalized to 16 S rRNA transcript levels, and change in expression level calculated as fold-change compared with minimal media, glucose cultures.

### Cell localization studies

Potential surface-located enzymes (containing either a predicted lipoprotein signal sequence or uncleaved TM anchor) encoded by the *B. ovatus* xylan PULs were initially identified using the LipoP 1.0 (http://www.cbs.dtu.dk/services/LipoP/) and SigP 4.1 (http://www.cbs.dtu.dk/services/SignalP/) servers. Note, the database N-term methionine for BACOVA_03425 GH43 is mis-annotated; the correct N-terminus actually starts 24aa downstream at the sequence MKN.

*Proteinase K treatment*. Cultures of *B. ovatus* (50 ml) were grown in minimal media on WAX (0.5% w/v) as a sole carbon source, to mid-exponential growth phase (OD_600_ ∼0.8). Cells were harvested by centrifugation and washed twice in 10 ml phosphate buffered saline pH 7.2 (PBS), before being resuspended in 2.5 ml of the buffer. The cells were split into four 0.5 ml aliquots. To three of the aliquots 2 mg ml^−1^ Proteinase K was added and incubated at 37 °C for up to 16 h, the fourth sample was left as an untreated control for 16 h. Following incubation with the proteinase the samples were centrifuged at 5,000 *g* for 10 min and the supernatant discarded. The cell pellets were resuspended in 1 ml PBS and remaining ProteinaseK precipitated by the addition of 200 μl trichloroacetic acid and incubation on ice for 30 min. The cells were pelleted by centrifugation and washed four times in 1 ml ice cold acetone. The cell pellets were resuspended in 250 μl Laemmli buffer and subjected to SDS–PAGE. Proteins were transferred to Whatman Protran BA 85 nitrocellulose membrane using a wet transfer system (BioRad Mini Trans-Blot). Proteins of interest were detected using anti-sera raised in rats (Eurogentec) against the corresponding recombinant form of the protein, diluted 1/1,000 or 1/2,000 in 1% milk powder in TBS buffer. The secondary antibody used was a chicken anti-rat conjugated to horseradish peroxidase (Santa Cruz) diluted 1/5,000 in 1% milk powder in TBS buffer. Antibodies were detected by chemi-luminescence using Biorad Clarity Western ECL Substrate.

*Immunofluorescence microscopy*. *B. ovatus* suspensions of mid-exponential phase cells (0.5 ml in PBS, produced as for Proteinase K treatment above) were fixed with an equal volume of 2 × formalin (9% formaldehyde in PBS), and rocked for 90 min at 25 °C in Eppendorf tubes. The cells were then pelleted by centrifugation for 3 min at 7,000 *g* and washed twice with 1 ml of PBS. The bacterial cell pellet was resuspended in 1 ml of blocking solution (2% goat serum, 0.02% NaN_3_ in PBS) and incubated at 4 °C for 16 h. After incubation cells were centrifuged again at 7,000 *g* and the supernatant discarded. For labelling, the bacteria were incubated with 0.5 ml of primary rat IgG (1/500 dilution of IgG in 1% milk powder in PBS, blocking solution) for 2 h at 25 °C. The cells were then pelleted, washed in 1 ml of PBS and resuspended in 0.4 ml goat anti-rat IgG Alexa-Fluor 488 (Sigma-Aldrich), diluted 1/500 in blocking solution and incubated 1 h at 25 °C in the dark. The cells were again pelleted, washed with PBS, resuspended in 1 ml of PBS containing ProLong Gold antifade reagent (Life Technologies). Aliquots (50 μl) of labelled bacterial cells were mounted onto glass slides and secured with coverslips. Fluorescence was visualized using a Leica SP2 UV microscope (Leica Microsystems, Heidelberg, GmbH) with × 63 NA1.32 lens. Alexa-Fluor 488-labelled bacteria were visualized under a UV view and compared with bright-field phase-contrast of the same image.

### Whole-cell assays

Whole-cell assays under aerobic conditions were used to determine surface enzyme activity uncoupled from glycan import. *B. ovatus* was grown in 5 ml minimal media containing WAX (0.5% w/v; activates both xylan PULs^4^), or glucose as the sole carbon source to mid exponential phase (OD_600_ ∼0.8). Cells were harvested by centrifugation at 4,000 *g* for 10 min at room temperature and washed in 5 ml PBS, pH 7.2, before being resuspended in 500 μl PBS. Cells (50 μl) were assayed against BGX, WAX and CX at 37 °C for 16 h. Assays were analysed by thin layer chromatography, 5 μl of each sample was spotted onto silica plates and resolved in butanol/acetic acid/water buffer (2:1:1). The plates were dried and carbohydrates visualized by orcinol/sulphuric acid heated to 70 °C for 10 min.

### Glycan-binding studies

The binding of proteins to their glycan ligands was quantified by isothermal titration calorimetry (ITC), as described previously^59^. Titrations were carried out in 50 mM Na-HEPES buffer, pH 7.5 at 25 °C. The reaction cell contained protein at 50–100 μM, while the syringe contained either the oligosaccharide at 1–10 mM or the polysaccharide at 3–10 mg ml^−1^. Integrated heats were fit to a single-site model using Microcal Origin v7.0 to derive *n*, *K*_*a*_, and Δ*H* values.

### Glycoside hydrolase assays

*Spectrophotometric assays*. The activity of the GH31 α-xylosidase and GH3 and GH43 β-xylosidases against xylans and xylooligosaccharides were quantified by the continuous monitoring of xylose release using a D-xylose detection kit (Megazyme International). The reaction was carried out at 37 °C in 50 mM sodium phosphate buffer, pH 7.0 containing 2 mM MgCl_2_, 1 mM ATP and 1 mM NAD^+^, excess concentrations of linked enzymes (hexokinase, xylose mutarotase and β-xylose dehydrogenase) and 1 mg ml^−1^ BSA. The amount of NADH formed in this reaction is stoichiometric with the amount of D-xylose released and NADH levels were monitored at 340 nm using an extinction coefficient of 6,220 M^−1^ cm^−1^. The xyloglucan heptasaccharide and linear xylooligosaccharide substrates used in this assay were purchased from Megazyme International. The glucurono-xylooligosaccharides, which were also used as substrates, were generated as described previously^37^. In brief, BGX was digested with CjXyn10A or NpXyn11A to generate UXX or XUXX, respectively, as the main charged oligosaccharides. These were purified from neutral xylooligosaccharides using Dowex chromatography and freeze dried. For kinetic assays of the GH43 α-L-arabinofuranosidases and GH67 α-D-glucuronidase, the L-arabinose/D-galactose assay and D-glucuronic acid assay kits (Megazyme International) were used, respectively^37^. The rye arabinoxylan used to assay the arabinofuranosidases was from Megazyme International.

The activity of the GH10 and GH30 endo-xylanases against BGX was determined in 50 mM sodium phosphate buffer, pH 7.0 at 37 °C containing an appropriate concentration of the polysaccharide and 1 mg ml^−1^ BSA. Reactions were incubated at 37 ^o^C and at regular time intervals 500 μl aliquots were removed and the amount of reducing sugar was quantified using the dinitrosalicylic acid reagent (Sigma Aldrich) at 575 nm and a standard curve of xylose in the reaction conditions used. HPAEC-PAD was used to determine the profile of the xylooligosaccharides generated by the endo-xylanases from these reactions and to measure the relative activity of the GH10 enzymes against specific xylooligosaccharides.

To measure the activity of the GH95 enzymes, the L-fucose and L-galactose products released from 2’-fucosyllactose (Carbosynth) and CX, respectively, were monitored by HPAEC-PAD. The reactions were carried out in 20 mM sodium phosphate buffer, pH 7.0 at 37 °C. In brief substrates were incubated with an appropriate concentration of each enzyme (10 nM to 5 μM). Aliquots were removed at regular intervals for up to 1 h and, after boiling for 10 min to inactivate the enzyme, the amount of the product was quantified by comparison to known standards. The activity of wild type and mutant *Bo*GH95 (10 μM final enzyme in 20 mM sodium phosphate buffer, pH 7.0 at 37 °C) against 2-chloro-4-nitrophenyl α-L-fucose (Carbosynth) was determined by continuously monitoring the release of 2-chloro-4-nitrophenol product at 400 nm.

The molar concentration of polysaccharide available to exo acting enzymes was determined by incubating the glycans at 1 mg ml^−1^ with a large excess of enzyme for 1 h to ensure the reaction goes to completion. The molar concentration of product at this point was then determined using the appropriate monosaccharide detection kit (detailed above). This equates to the molar concentration of total available substrate, thus enabling mg ml^−1^ of substrate to be converted to molarity.

Where *k*_cat_/*K*_*m*_ alone is reported this was determined by monitoring the initial rate at a range of substrate concentrations significantly below the *K*_*m*_ such that a linear plot of V_0_ vs [S] was obtained. Thus, the initial rate of substrate hydrolysis gives a direct readout of *k*_cat_/*K*_*m*_ using the equation *k*_cat_/*K*_*m*_=V_0_/[S]*[E].

*High-performance anion-exchange chromatography*. Products generated by glycoside hydrolases used in the study were analysed using an analytical CARBOPAC^TM^ PA-100 anion-exchange column (Dionex) equipped with a guard column. The system had a loop size of 100 μl and was run at a flow rate of 1 ml min^−1^ at a pressure of ∼2,300 lbf in^−2^. Carbohydrates were detected by pulsed amperometric detection (PAD) with electrode settings of E_1_=+0.05, E_2_=+0.6 and E_3_=−0.6. The elution conditions were 0–10 min, 100 mM NaOH; 10–25 min, 100 mM NaOH with a 0–75 mM sodium acetate gradient; and 25–35 min, 100 mM NaOH containing 500 mM sodium acetate. For analysis of the very large oligosaccharides produced by the GH98 xylanase a 0-100% sodium acetate gradient from 0 to 90 mins was used. The standards used to identify the chromatographic peaks were arabinose, xylose, xylobiose, xylotriose, xylotetraose, xylopentaose and xylohexaose.

*Analysis of the GH98 (BACOVA_03433) products reduced with sodium borohydride*. To analyse the specificity of BACOVA_03433, the recombinant protein (5 μM final) was incubated with 20 mg of CX resuspended in 0.5 ml 50 mM sodium phosphate buffer, pH 7.0 at 37 °C for 24 h. The same amount of CX was incubated separately without the enzyme as a control. Both samples were then incubated overnight with NaBH_4_ (10 mg per sample) in the presence of NH_4_OH, pH 10.8 at 25 °C. After incubation, samples were freeze-dried, resuspended in 500 μl of water and loaded separately on Bio-gel P2 size-exclusion columns (Bio-Rad laboratories Inc.). Water was applied to the column (0.5 ml min^−1^) using a Pharmacia CKB peristaltic pump to elute carbohydrate products. A 4 μl aliquot of each of the 120 fractions (2 ml) was spotted on silica gel TLC plates, which were developed in butanol:acetic acid:water 2:1:1 and carbohydrate products detected by spraying with 0.5% (w/v) orcinol in 10% sulphuric acid and heating to 70 °C for 10 min. Those containing oligosaccharide were pooled together and dried using a speed-vac. Each sample (enzyme and control) was then resuspended in 20 μl of water and hydrolysed with 0.3 M HCl for 3 h at 100 °C. The products of acid hydrolysis were neutralized with 1 M NaOH and analysed by HPAEC using standard methodology (see above). The identity of the reduced sugar product was determined by comparison to known standards (galactitol and xylitol).

### X-ray crystallography

BACOVA_03438 GH95, at 10 mg ml^−1^, was crystallized from 160 mM calcium acetate, 80 mM sodium cacodylate pH 6.5, 14.4% w/v polyethylene glycol 8,000 and 20% v/v glycerol using the vapour diffusion sitting drop method. Crystals of BACOVA_03438 in complex with L-galactose were obtained by preparing protein:monosaccharide mixtures at 10 mg ml^−1^: 300 mM and mixing 1:1 or 2:1 with the reservoir solution grown at 20 °C. Crystals were mounted in mother liquor and cryocooled in liquid N_2_. Data for BACOVA_03438 were collected at Diamond Light Source, UK, beamline I02, to 2.81 Å resolution. Data were processed and integrated with XDS and scaled using Aimless^60^,^61^. All other computing used the CCP4 suite of programs^62^. The space group was determined to be *P2*_*1*_ *2*_*1*_ *2*_*1*_ with 2 molecules in the asymmetric unit (giving a Matthews coefficient of 2.61 Å^3^ Da^−1^ and a solvent content of 53%). The structure of BACOVA_03438 was solved by molecular replacement in MOLREP using pdb file with accession code 2RDY as the search model. The model underwent cycles of model building in COOT^63^ and refinement in REFMAC^64^. Five percent of the observations were used to monitor the progress of refinement. The model was submitted to the PDB_REDO server to improve final model quality^65^. The model was validated using Molprobity^66^. The crystal and data collection statistics are reported in Supplementary Table 12.

### Genetic manipulation of *Bacteroides ovatus*.

Knockout strains lacking either PUL-XylL, PUL-XylS or both PULs were created using counterselectable allelic exchange as described previously^30^. The inactive mutant GH98 (BACOVA_03433) in pET21 lacking both catalytic residues was made using the Quikchange site directed mutagenesis kit (Agilent) following the manufacturer’s protocol (Supplementary Table 10). The mutated gene was then cloned into pExchange and integrated into the genome of *B. ovatus* using the same counterselectable allelic exchange methodology as for the PUL knockouts^30^. Signature-tagged strains of wild type and the ΔGH98 mutant, for determining the ratios of each during co-culture by qPCR, were made by integration of unique sequences into one of two *att* sites in the *Bacteroides ovatus* genome through homologous recombination of pNBU2 conjugated into the *Bacteroides* from S17-1 λpir *E. coli* as described previously^3^. Following positive selection on BHI-agar contain gentamycin (200 μg ml^−1^) and tetracycline (1 μg ml^−1^), genomic DNA was extracted (QIAamp DNA Mini Kit; Qiagen) and PCR with primers flanking each possible *att* insertion site was used to select for *Bacteroides* strains that had successfully integrated the tag sequence. Primers used for all genetic manipulations are shown in Supplementary Table 13.

### Sharing assays

*Bifidobacterium adolescentis* and *Bacteroides ovatus* cells were grown anaerobically to mid/late-log phase in CNM (Sigma-Aldrich) and TYG rich media, respectively. Cells were washed in PBS and used, in approximately equal proportions, to inoculate Minimal Medium (based on medium devised by Van der Meulen *et al.*^58^) containing 0.5% (w/v) BGX, WAX or CX as the sole carbon source. During co-culture of *Bacteroides* and *Bifidobacterium* aliquots were taken over 18 h. Serial dilutions of the samples were plated onto CNM agar and incubated anaerobically for 48 h at 37 °C to obtain CFU of each sample. Single colonies were picked and replica plated onto CNM and CNM with 200 μg ml^−1^ gentamycin to differentiate between *Bacteroides* and *Bifidobacterium* as *Bacteroides* spp. are resistant to this antibiotic. The percentage of each bacterium present was used, in conjunction with total CFUs was used to calculate the CFU of each.

To compare ratios of the ΔGH98 when grown in co-culture with wild-type *B. ovatus* on CX, 5 ml minimal medium containing 0.5% CX was inoculated with tagged ΔGH98 and wild type overnights grown on TYG in a 50:50 ratio (estimated by OD_600nm_). Samples (0.5 ml) were taken during growth, 50 μl of which was serial diluted and plated onto to BHI agar, while genomic DNA was isolated from the remaining cells. gDNA samples were subject to quantitative PCR (Roche LightCycler 480 System; see above) using tag-specific primers (Supplementary Table 13) to give relative quantities of the mutant and wild-type cells in the culture. The ratio obtained by qPCR with total CFU counts was used to deduce CFU of mutant and wild type at the different phases of growth.

To analyse the growth of *B.* a*dolescentis* on digested xylans, *Bifidobacterium* minimal medium containing with 0.5% WAX, BGX or CX that had been digested to completion with either recombinant BACOVA_04390 GH10 for WAX and BGX, or for CX with BACOVA_03433 GH98 alone, or all surface-located enzymes from PUL-XylL (BACOVA_03419 GH3, 03421 GH43, 03432 GH30 and 03433 GH98), was inoculated with a *B.* a*dolescentis* overnight that had been grown on rich medium (CNM). Aliquots were taken at different phases of growth, serial diluted and plated onto CNM agar to determine CFU at each growth phase.

### Comparative genomics analysis

PULs similar to PUL-XylS and PUL-XylL were searched for in 283 Bacteroidetes genomes (complete list provided in Supplementary Table 8). The identification of similar PULs was based on PUL alignments. Gene composition and order of Bacteroidetes PULs were first computed using the PUL predictor described in PULDB^67^. Then, in a manner similar to amino-acid sequence alignments, the predicted PULs were aligned to PUL-XylS and PUL-XylL according to their modularity as proposed in RADS/RAMPAGE method^68^. Modules taken into account include CAZy families, sensor-regulators and *susCD*-like genes. Finally, PUL boundaries and limit cases were refined by BLASTP-based analysis.

## Additional information

**Accession codes:** Coordinates and structure factors for BACOVA_03438 have been deposited in the RCSB Protein Data Bank under accession code 4UFC.

**How to cite this article:** Rogowski, A. *et al.* Glycan complexity dictates microbial resource allocation in the large intestine. *Nat. Commun.* 6:7481 doi: 10.1038/ncomms8481 (2015).

## Supplementary Material

Supplementary Information Supplementary Figures 1-9, Supplementary Tables 1-13 and Supplementary References
