# Corrigendum: Glycan complexity dictates microbial resource allocation in the large intestine

**DOI:** 10.1038/ncomms10705

**Published:** 2016-02-05

**Authors:** Artur Rogowski, Jonathon A. Briggs, Jennifer C. Mortimer, Theodora Tryfona, Nicolas Terrapon, Elisabeth C. Lowe, Arnaud Baslé, Carl Morland, Alison M. Day, Hongjun Zheng, Theresa E. Rogers, Paul Thompson, Alastair R. Hawkins, Madhav P. Yadav, Bernard Henrissat, Eric C. Martens, Paul Dupree, Harry J. Gilbert, David N. Bolam

Nature Communications 6 Article number: 748110.1038/ncomms8481 (2015); Published 06262015; Updated 02052016

The financial support for the work described in this Article was not fully acknowledged. The Acknowledgements should have included the following:This work was supported in part by a grant to H.J.G., B.H. and N.T. (European Union's Seventh Framework Program (FP/2007/2013)/European Research Council (ERC) Grant Agreement 322820).

