# Supplementary material for: Glycan complexity dictates microbial resource allocation in the large intestine
Source: Nat Commun. 2015 Jun 26;6:7481. doi: 10.1038/ncomms8481 (PMC4491172; doi:10.1038/ncomms8481)
Supplement: Supplementary Information — Supplementary Figures 1-9, Supplementary Tables 1-13 and Supplementary References [file ncomms8481-s1.pdf]

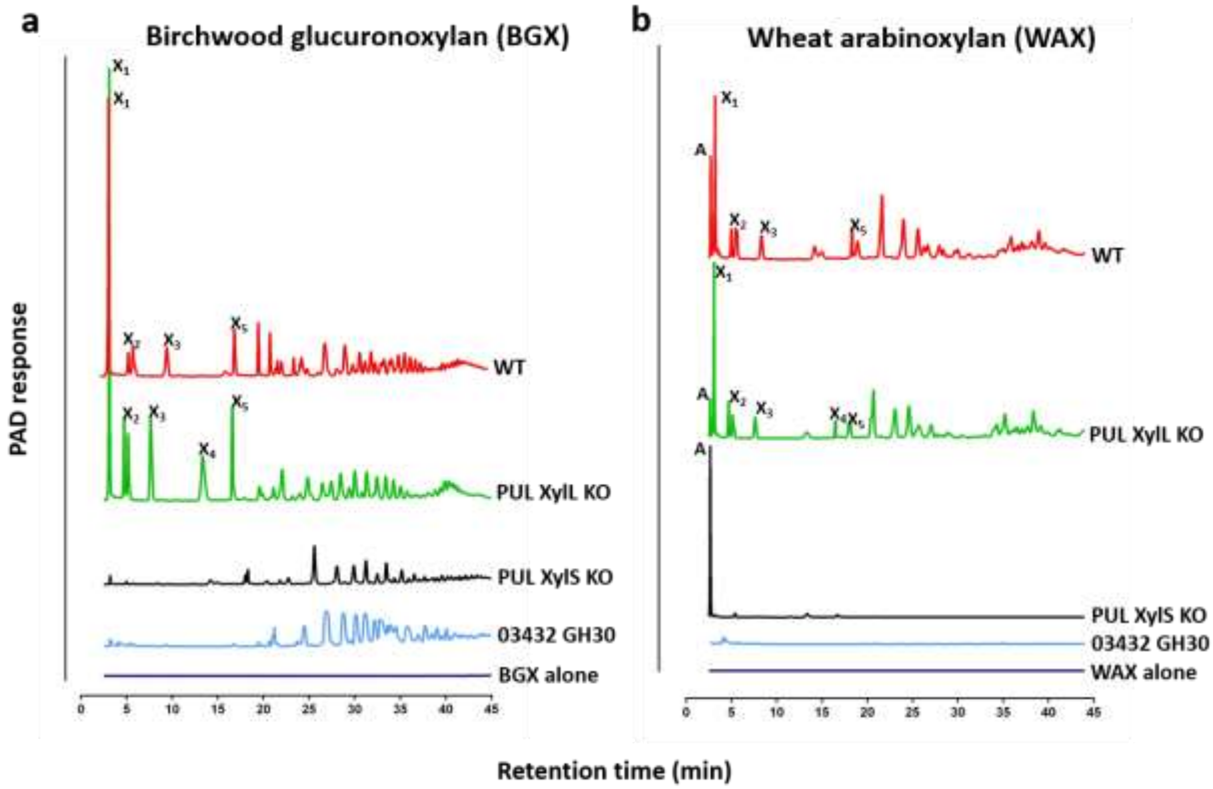


**Supplementary Figure 1. The degradation of xylans by *B. ovatus* surface located enzymes.** Wild type and mutants (whole PUL knockouts; PUL XylS and PUL XylL KO) of *B. ovatus* were grown on WAX or CX. The cells were then washed and incubated for 5 h at 37 °C with WAX (***a***) or BGX (***b***) under aerobic conditions and were thus metabolically inactive (no active glycan import). In addition, the recombinant form of the GH30 enzyme (BACOVA_03432) was incubated with BGX or WAX for 14 h. The reaction products were analysed by HPAEC-PAD and identified by reference to the following standards; arabinose (A) xylose (X1), xylobiose (X2), xylotriose (X3), xylotetraose (X4) and xylopentaose (X5).

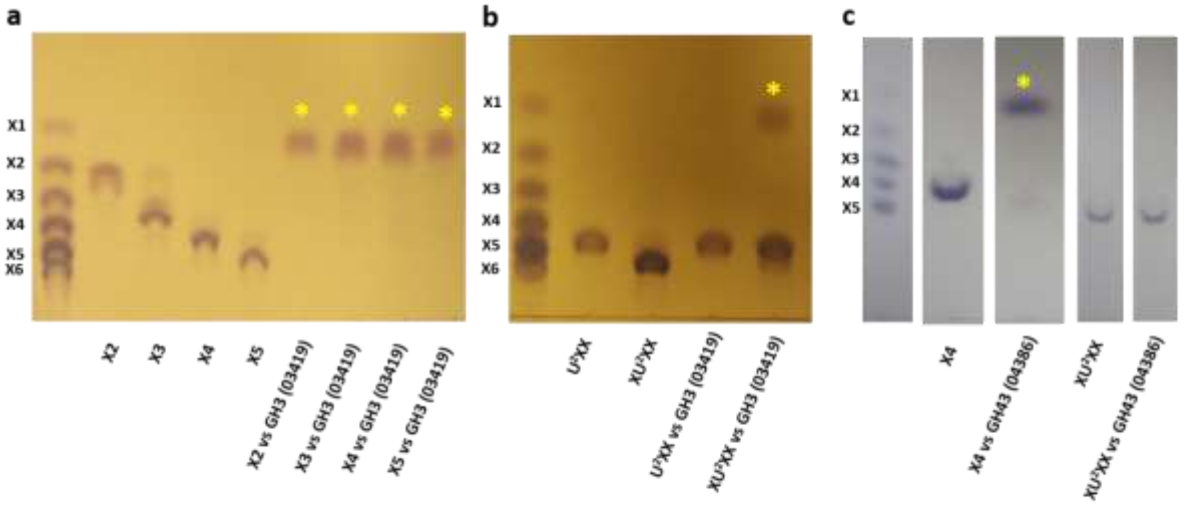


**Supplementary Figure 2. Activity of the β-xylosidases BACOVA_03419 and BACOVA_04386.** ***a,*** The surface GH3 β-xylosidase BACOVA_03419, at 1 μM, was incubated with 1mM β1,4-linked xylooligosaccharides for 16 h in 50 mM sodium phosphate buffer, pH 7.0, at 37 °C. ***b,*** BACOVA_03419 incubated with 1 mM $U^2$XX and X$U^2$XX (X is an undecorated xylose and $U^2$ signifies a xylose decorated with an α-D-(Me)glucuronic acid at O2[1]). ***c,*** The cytoplasmic GH43 β-xylosidase, BACOVA_04386, was incubated with linear xylotetraose and X$U^2$XX. The reactions conditions used in panels ***b*** and ***c*** were as described for ***a***. X1-X5 are xylooligosaccharide standards and the xylose released is indicated by a yellow asterisk.

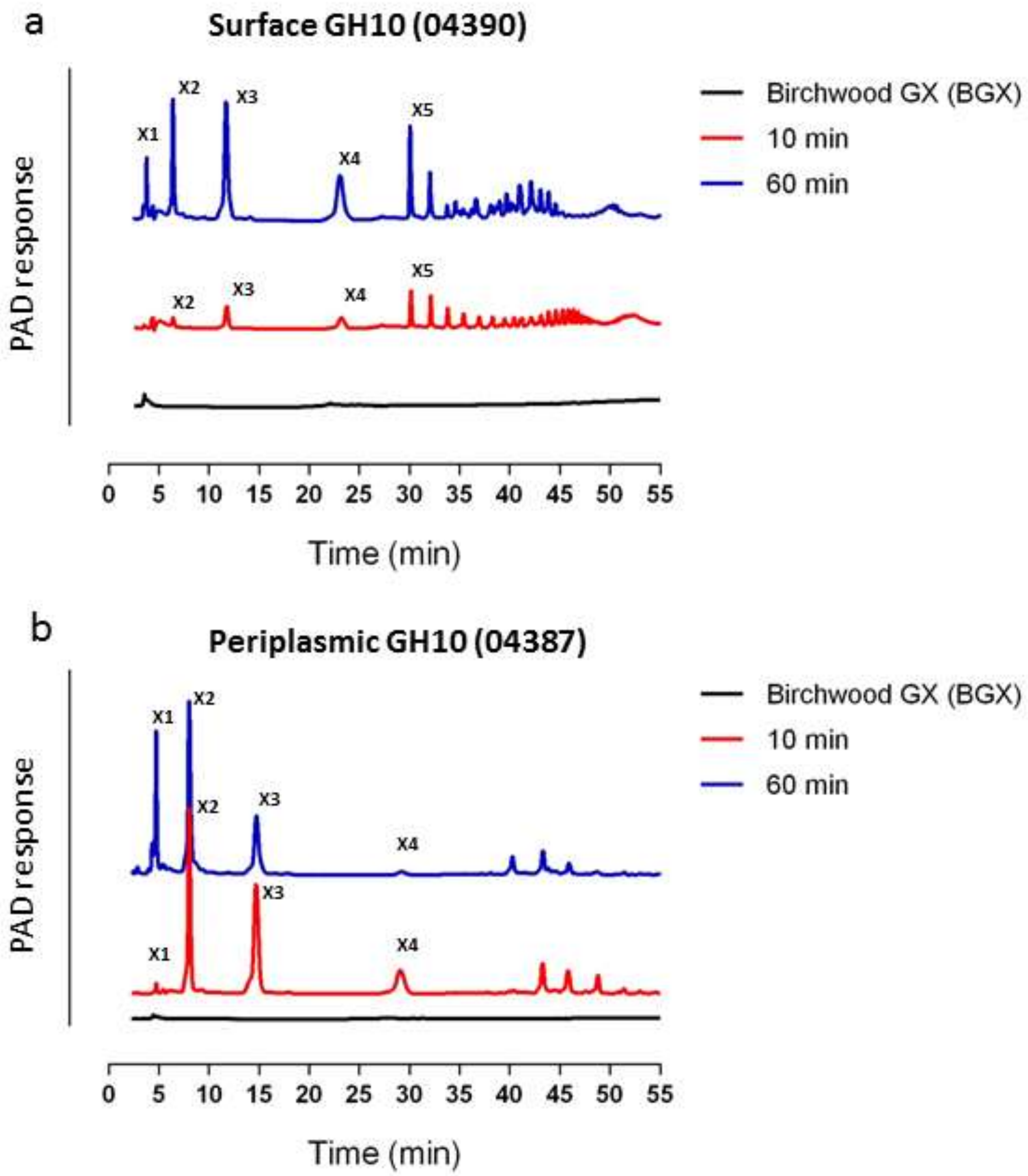


**Supplementary Figure 3. The product profile of the surface and periplasmic GH10 xylanases against glucuronoxylan.** The surface (**a,** BACOVA_04390) and periplasmic (**b,** BACOVA_04387) GH10 xylanases at 200 nM were incubated with BGX (1% w/v) in 20 mM sodium phosphate buffer, pH 7.0, at 37 °C. The reaction products were subjected to HPAEC and identified by their co-migration with known xylooligosaccharide standards (X1-X5).

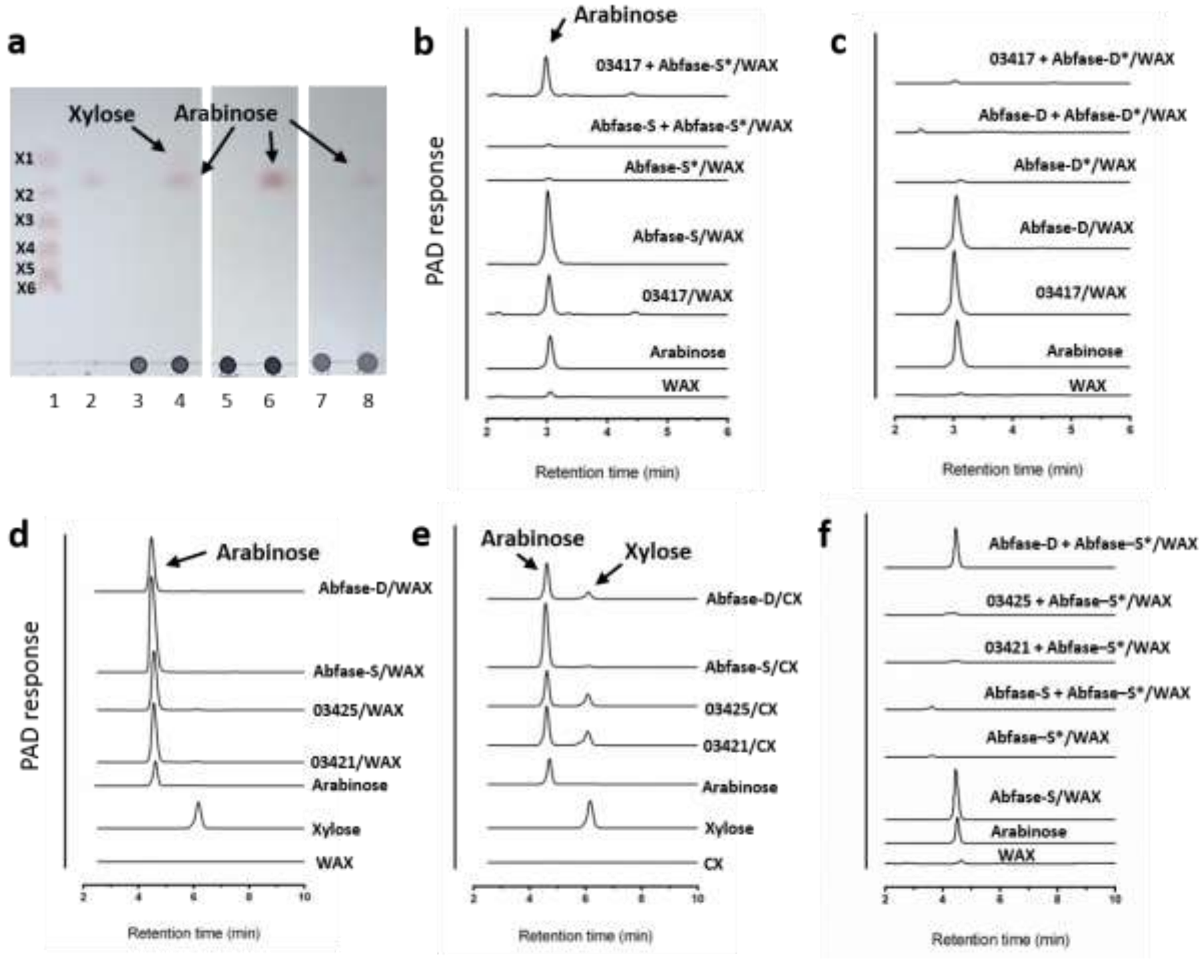


**Supplementary Figure 4. Activity of the GH43 α-L-arabinofuranosidases BACOVA_03417, BACOVA_03421 and BACOVA_03425 against polysaccharides**. The polysaccharide at 1% (w/v) was incubated with 1 μM of enzyme at 37 °C for 16 h in 50 mM sodium phosphate buffer, pH 7.0. ***a,*** The products of BACOVA_03417 were analysed by TLC. The lanes contained the following: 1, xylooligosaccharide standards with a d.p. of 1-6; 2, arabinose standard; 3, corn xylan (CX); 4, CX + BACOVA_03417; 5, wheat arabinoxylan (WAX); 6, WAX + BACOVA_03417; 7, sugar beet arabinan; 8, sugar beet arabinan + BACOVA_03417. ***b,*** HPAEC analysis of the products released from WAX by BACOVA_03417 enzyme together with an arabinofuranosidase from *C. japonicus*[2] that targets either O2 or O3 single arabinose decorations (defined as Abfase-S). Abfase-S*/WAX denotes WAX pretreated with Abfase-S to remove all single arabinose decorations followed by boiling and then dialysis so that only the polysaccharide remained. This material was then

incubated with either additional Abfase-S (Abfase-S + Abfase-S*/WAX; shows that all the single arabinose units had been removed) or BACOVA_03417 (03417 + Abfase-S*/WAX; shows that BACOVA_03417 does not target single arabinose substitutions). ***c,*** HPAEC analysis of the products released from WAX by BACOVA_03417 together with a *Humicola* arabinofuranosidase[3] that specifically targets arabinose units that are linked O3 to double substituted xylose residues (Abfase-D). Abfase-D*/WAX denotes WAX pretreated with Abfase-D to remove arabinose decorations from double substitutions, followed by boiling and then dialysis so that only the polysaccharide remained. This material was then incubated with either additional Abfase-D (Abfase-D + Abfase-D*/WAX; shows that all the double arabinose units had been removed) or BACOVA_03417 (03417 + Abfase-D*/WAX; shows that BACOVA_03417 also targets double arabinose substitutions as no product was released). ***d,*** and ***e,*** display HPAEC analysis of products generated by the single specific GH43 $\alpha$-L-arabinofuranosidases encoded by PUL-XylL, BACOVA_03421 and BACOVA_03425, against WAX and CX, respectively. ***f***, Shows that both BACOVA_03421 and BACOVA_03425 are single specific enzymes as neither releases arabinose from WAX after the polysaccharide has been pre-treated with *C. japonicus* Abfase-S (03421 + Abfase-S*/WAX and 03425 + Abfase-S*/WAX).

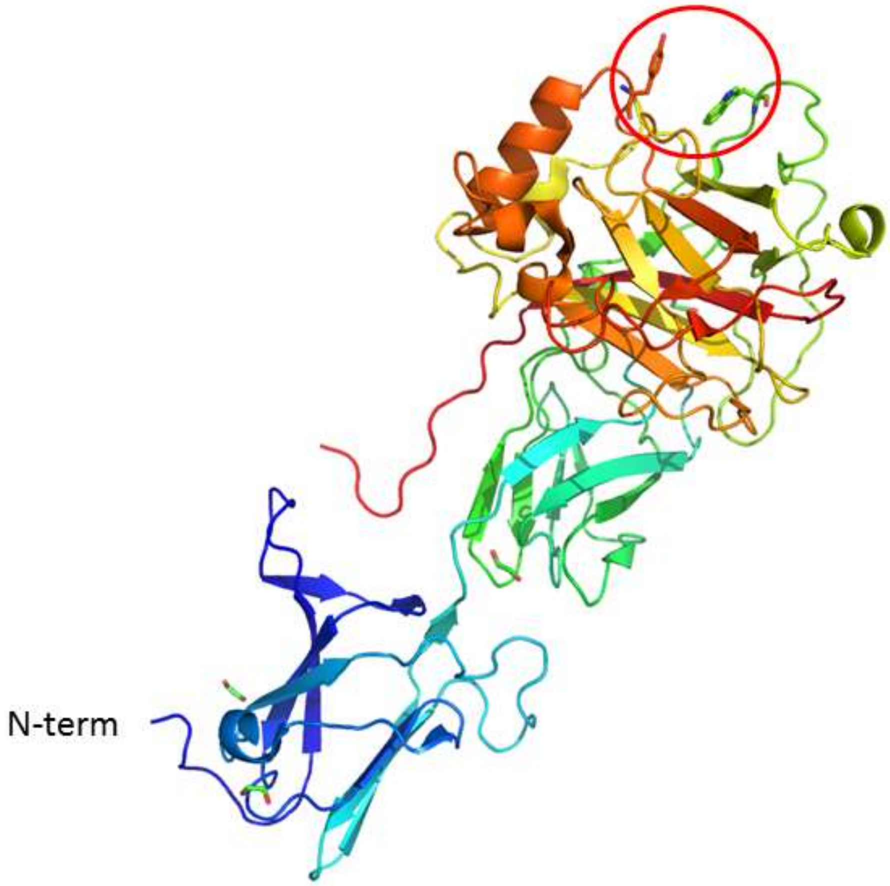


**Supplementary Figure 5. Structure of the surface xylan binding protein BACOVA_04391 (PDB 3ORJ).** The protein is displayed as a secondary structure schematic that is colour ramped from blue (N-terminus) to red (C-terminus). The three β-sandwich domains domains are coloured blue, green and orange/red. The likely xylan binding site with the opposing solvent exposed aromatic resides that are proposed to be important in ligand binding is circled in red, with the amino acid side chains shown in stick format. BACOVA_04391 is a lipoprotein and will thus be attached to the outer membrane via its N-term, providing significant spatial separation between the membrane and the xylan binding site.

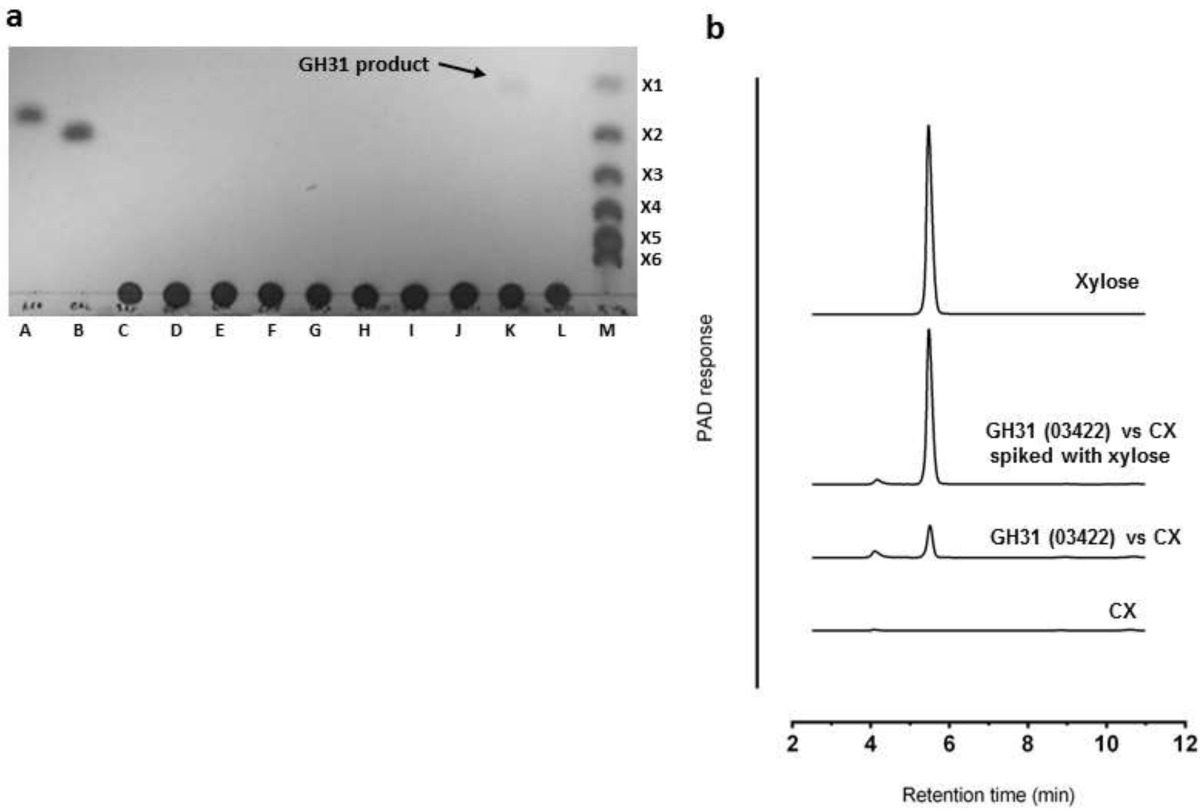


**Supplementary Figure 6. Activity of the GH31 enzyme BACOVA_03422.** ***a,*** TLC of rye AX (C, H), BGX (D, G) oat spelt AX (E, I), CX (F, K) and WAX (G, L) all at 5 mg/ml, incubated for 16 h in 20mM sodium phosphate, pH 7.0, at 37 $^{o}$C in the absence (lanes C-G) or presence (lanes H-L) of 1 μM BACOVA_03422 GH31. Standards were arabinose (lane A), galactose (lane B) and xylooligosaccharides with a d.p. of 1-6 (lane M). Only incubation of the enzyme with CX (lane K) released a reaction product, which co-migrated with xylose. ***b,*** The product derived from CX was spiked with xylose and analysed by HPAEC-PAD.

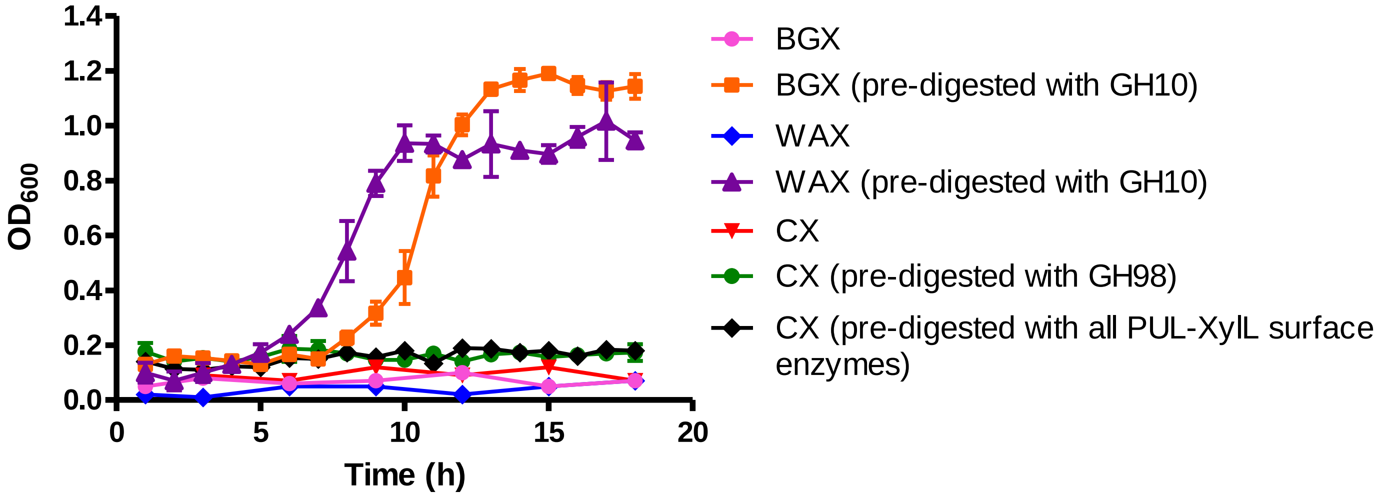


**Supplementary Figure 7. Growth of *Bifidobacterium adolescentis* on pre-digested and undigested xylans.** Pre-digested indicates the xylan was digested to completion with the surface enzymes from the two *B. ovatus* xylan PULs prior to addition to minimal media at 0.5% final. GH10 = BACOVA_04390 (PUL-XylS); GH98 = BACOVA_03433 (PUL-XylL). All surface enzymes from PUL-XylL = BACOVA_03419 GH3, BACOVA_03421 GH43, BACOVA_03432 GH30 and BACOVA_03433 GH98. Data are averages and standard deviations of 3 separate growths.

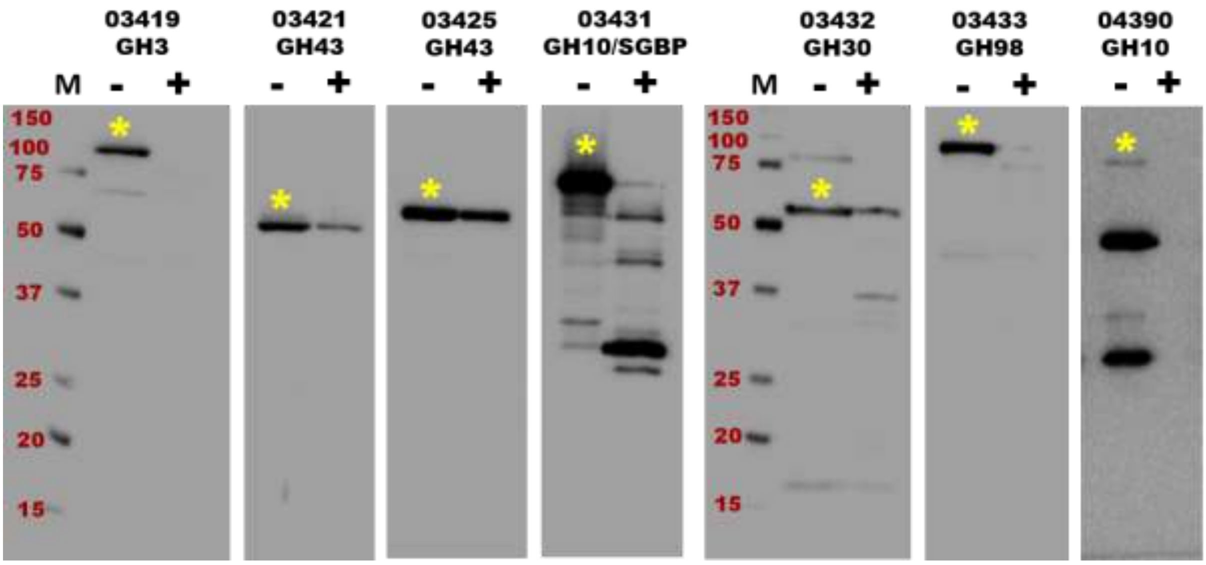


**Supplementary Figure 8. Western blots of *B. ovatus* cells grown on WAX, either untreated with proteinase K (-) or incubated with 2 mg/ml proteinase K for 16 h (+).** The blots were probed with antibodies against the *B. ovatus* enzymes indicated. The yellow asterisk indicates the band at the predicted molecular weight of the target protein. Note that for BACOVA_04390 the major bands visible appear to be processed forms of the full length enzyme. Lanes labelled M are the molecular weight markers (sizes shown in kDa).

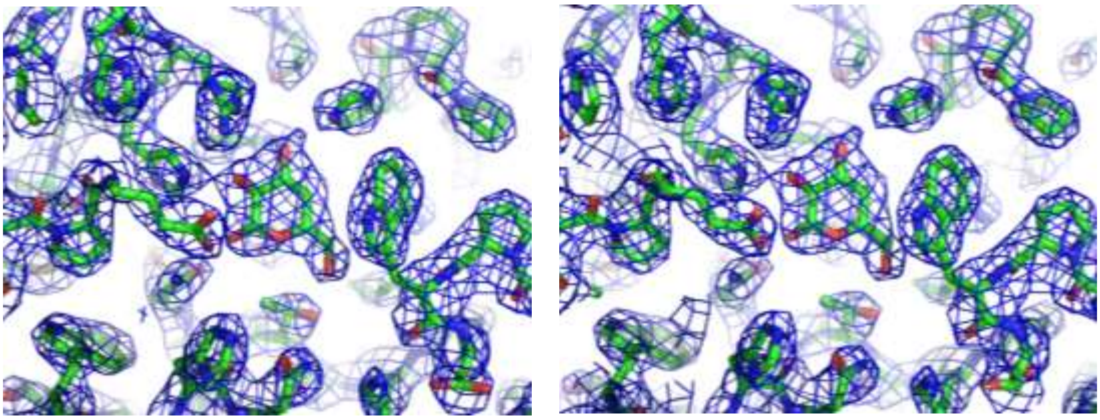

**Supplementary Figure 9. Stereo image of a portion of the electron density map of BACOVA_03438 GH95.** The PDB model is represented as sticks with carbon atoms in green, oxygen atoms in red and nitrogen atoms in blue.

**Supplementary Table 1. Kinetics of PUL-XylL and PUL-XylS β-xylosidases against linear and substituted xylooligosaccharides**

| Substrate | GH3 (03419) | GH43 (04386) |
|---|---|---|
| | $k_{cat}/K_m$ ($s^{-1}$ $mM^{-1}$) | $k_{cat}/K_m$ ($s^{-1}$ $mM^{-1}$) |
| Xylobiose ($X_2$) | 0.06 (±3.3x$10^{-5}$)[a] | 0.4 (±8.5x$10^{-4}$) |
| Xylotriose ($X_3$) | 0.04 (±1.4x$10^{-4}$) | 2.2 (±7.3x$10^{-3}$) |
| Xylotetraose ($X_4$) | 0.06 (±2.3x$10^{-5}$) | 2.4 (±5.1x$10^{-5}$) |
| Aldotriuronic acid ($U^2XX$)[b] | ND[c] | ND |
| Aldopentauronic acid ($XU^2XX$) | 0.14 (±1.7x$10^{-4}$) | ND |
| Arabinotetrasaccharide ($XA^3X$)[d] | 0.03 (±2.9x$10^{-5}$) | ND |

[a]Values are from a single experiment and errors are derived from the fit of the linear regression to the data.
[b]$U^2$ = (Me)GlcA substituted on the O2 of xylose[2].
[c]ND = no activity detected.
[d]$A^3$ = arabinose substituted on the O3 of xylose.

**Supplementary Table 2. Kinetics of PUL-XylS and PUL-XylL GH10 and GH30 endo-xylanases against xylan and xylooligosaccharides**

| Substrate | GH30 (03432) | GH10 (03431) | GH10 (04387) | GH10 (04390) |
|---|---|---|---|---|
| | $k_{cat}/K_m$ ($s^{-1}$ $mg^{-1}$ ml) | $k_{cat}/K_m$ ($s^{-1}$ $mg^{-1}$ ml) | $k_{cat}/K_m$ ($s^{-1}$ $mg^{-1}$ ml) | $k_{cat}/K_m$ ($s^{-1}$ $mg^{-1}$ ml) |
| Birch glucuronoxylan (BGX) | 3.8 (±0.13)[a] | ND | 150 (±16.7) | 4.7 (±0.39) |
| | | | | |
| | | | **Relative activity** | **Relative activity** |
| Xylotriose ($X_3$) | ND[b] | ND | 0.001 | UQ[c] |
| Xylotetraose ($X_4$) | ND | ND | 0.15 | 0.0003 |
| Xylopentaose ($X_5$) | ND | ND | 1.1 | 0.004 |
| Xylohexaose ($X_6$) | ND | ND | 1 | 0.03 |

[a]Values are from a single experiment and errors are derived from the fit of the linear regression to the data.
[b]ND = no activity detected.
[c]UQ = activity too low to quantify.

**Supplementary Table 3. ITC analysis of the SusD-likes and surface glycan binding proteins from the xylan PULs binding to xylans and xylooligosaccharides**

| Protein | Ligand | Ka ($M^{-1}$) | ΔG (kcal $mol^{-1}$ ) | ΔH (kcal $mol^{-1}$) | TΔS (kcal $mol^{-1}$) | n |
|---|---|---|---|---|---|---|
| **PUL-XylS** | | | | | | |
| SusD-like (BACOVA_04392) | BGX[a] | $3.7 \times 10^3$ (±0.5)[b] | -4.9 | -15.4 (±1.2) | -10.5 | 1.0[c] |
| | WAX | $2.8 \times 10^3$ (±0.2) | -4.7 | -10.2 (±0.4) | -5.5 | 1.0 |
| | CX | NB[d] | - | - | - | - |
| | X6 | $8.0 \times 10^2$ (±1.3) | -4.0 | -[e] | | - |
| | ≤X5 | NB | - | - | - | - |
| SGBP[f] (BACOVA_04391) | BGX | $2.0 \times 10^4$ (±1.2) | -5.5 | -10.8 (±1.4) | -6.3 | 1.0 |
| | WAX | $1.5 \times 10^4$ (±0.6) | -5.7 | -10.2 (±1.3) | -4.5 | 1.0 |
| | CX | NB | - | - | - | - |
| | X6 | $1.8 \times 10^4$ (±0.3) | -5.8 | -11.2 (±3.8) | -5.4 | 0.85 (±0.2) |
| | X5 | $9.1 \times 10^3$ (±0.7) | -5.4 | -8.9 (±0.2) | -3.5 | 1.0 (±0.2) |
| | X4 | $1.3 \times 10^3$ (±0.1) | -4.2 | -[e] | - | - |
| | ≤X3 | NB | | | | |
| **PUL-XylL** | | | | | | |
| SusD-like_1 (BACOVA_03427)[g] | BGX | $8.4 \times 10^4$ (±0.9) | -6.7 | -9.2 (±0.5) | -2.9 | 1.0 |
| | WAX | NB | - | - | - | - |
| | CX | $1.5 \times 10^5$ (±0.1) | -7.1 | -8.1 (±0.2) | -1.0 | 1.0 |
| | ≤X6 | NB | - | - | - | - |
| SGBP (BACOVA_03431; inactive GH10) | BGX | $1.3 \times 10^4$ (±0.1) | -5.6 | -9.9 (±0.8) | -4.3 | 1.0 |
| | WAX | NB | - | - | - | - |
| | CX | $1.5 \times 10^4$ (±0.1) | -5.7 | -17.3 (±0.8) | -11.6 | 1.0 |
| | ≤X6 | NB | - | - | - | - |

[a]BGX = birchwood glucuronoxylan, WAX = wheat arabinoxylan, CX = corn glucuronoarabinoxylan.
[b]Data are averages and standard deviations of between two and three independent titrations.
[c]The data with xylans was fit with a molar concentration of ligand such that n=1.
[d]NB – no binding detected. [e]Affinity too low to obtain accurate thermodynamic data. [f]SGBP – surface glycan binding protein. [g]The other SusD-like from PUL-XylL, BACOVA_03429, could not be tested as the protein did not express in a recombinant form.

**Supplementary Table 4. Kinetics of PUL-XylL double and single specific α-L-arabinofuranosidases against arabinoxylans**

| Enzyme | substrate | $k_{cat}$ ($s^{-1}$) | $K_m$ (mM) | $k_{cat}/K_m$ ($s^{-1}$ $mM^{-1}$) |
|---|---|---|---|---|
| **GH43 (03417 - double)** | Wheat arabinoxylan (WAX) | 3.3 (±0.15)[a] | 0.22 (±0.004) | 15 |
| **GH43 (03421 - single)** | WAX | 7.0 (±0.6) | 0.13 (±0.005) | 54 |
| **GH43 (03425 - single)** | WAX | 11.0 (±0.8) | 0.18 (±0.006) | 61 |
| **GH43 (03417 - double)** | Corn xylan (CX) | 2.7 (±0.2) | 0.09 (±0.002) | 30 |
| **GH43 (03421 - single)** | CX | 5.8 (±0.4) | 0.95 (±0.003) | 6 |
| **GH43 (03425 - single)** | CX | 9.3 (±1.1) | 2.9 (±0.01) | 3 |

[a]Values are averages and standard deviations of three independent experiments.

**Supplementary Table 5. Kinetics of PUL-XylS GH67 vs *C. japonicus* GlcA67A[4] α-glucuronidases**

| Substrate | ***Bo*GH67 (04385)** | ***Cj*GlcA67A** |
|---|---|---|
| | $k_{cat}/K_m$ ($s^{-1}$ $mM^{-1}$) | $k_{cat}/K_m$ ($s^{-1}$ $mM^{-1}$) |
| Aldotetrauronic acid ($U^2XX$) | 270 (±0.23)[α] | 320 (±0.28) |

[α]Values are from a single experiment and errors are derived from the fit of the linear regression to the data.

**Supplementary Table 6. Kinetics of PUL-XylL GH31 vs *C. japonicus* Xyl31A[5] α-xylosidases**

| Substrate | ***Bo*GH31 (03422)** | ***Cj*Xyl31A** |
|---|---|---|
| | $k_{cat}/K_m$ ($s^{-1}$ $mM^{-1}$) | $k_{cat}/K_m$ ($s^{-1}$ $mM^{-1}$) |
| Xyloglucan heptasaccharide (XXXG) | 0.02 (±0.003)[α] | 403 (±0.26) |
| Corn xylan (CX) | 58 (±0.02) | 0.65 (±0.003) |

[α]Values are from a single experiment and errors are derived from the fit of the linear regression to the data.

**Supplementary Table 7. Activity of wild type and mutant forms of PUL-XylL α-L-galactosidase vs *B. bifidum* α-L-fucosidase AfcA[6] GH95s**

| Substrate | WT *Bo*GH95 (03438) | *Bb*GH95 (AfcA) | *Bo*GH95 E501A | *Bo*GH95 T370H |
|---|---|---|---|---|
| | $k_{cat}/K_m$ ($s^{-1}$ $mM^{-1}$) | $k_{cat}/K_m$ ($s^{-1}$ $mM^{-1}$) | | |
| Corn xylan (CX) | 26 (±0.51)[α] | 0.02 (±0.006) | ND[b] | ND |
| | | | | |
| | **Relative activity** | **Relative activity** | | **Relative activity** |
| 2'fucosyl-lactose[c] | 1 | 3700 | - | - |
| 2-chloro-4-nitrophenyl α-L-fucose[d] | 1 | - | - | 0.13 |

[α]Values are from a single experiment and errors are derived from the fit of the linear regression to the data.
[b]ND = no activity detected.
[c] Values for fucosyl lactose are relative activity at 0.25mM sugar as the $K_m$ of the *Bb*GH95 for this substrate was too low to measure a valid $k_{cat}/K_m$.
[d] Values for 2-chloro-4-nitrophenyl α-L-fucose are relative activity vs 5mM substrate.

**Supplementary Table 8. List of Bacteroidetes species searched for the presence of XylS and XylL PULs**

| Organism name | NCBI taxonomy ID | IMG/M HMP project ID |
|---|---:|---:|
| *Aequorivita sublithincola DSM 14238* | 746697 | 2509276063 |
| *Algoriphagus marincola DSM 16067* | 1120966 | 2523533625 |
| *Algoriphagus vanfongensis DSM 17529* | 1120968 | 2523231017 |
| *Alistipes finegoldii DSM 17242* | 679935 | 2509601035 |
| *Alistipes indistinctus YIT 12060* | 742725 | 2513237277 |
| *Alistipes onderdonkii DSM 19147* | 1120974 | 2517572213 |
| *Alistipes putredinis DSM 17216* | 445970 | 641736205 |
| *Alistipes shahii WAL 8301* | 717959 | 650377904 |
| *Alistipes sp. HGB5* | 908612 | 2512047053 |
| *Alloprevotella rava F0323* | 679199 | 2513237313 |
| *Anaerophaga thermohalophila DSM 12881* | 886379 | 2515154051 |
| *Anaerophaga thermohalophila str. Valhall* | 1144273 | 2515154050 |
| *Arenibacter certesii DSM 19833* | 1121011 | 2523231023 |
| *Bacteroides barnesiae DSM 18169* | 1121094 | 2517572120 |
| *Bacteroides caccae ATCC 43185* | 411901 | 640963023 |
| *Bacteroides cellulosilyticus DSM 14838* | 537012 | 643886111 |
| *Bacteroides clarus YIT 12056* | 762984 | 651324010 |
| *Bacteroides coprocola DSM 17136* | 470145 | 642791613 |
| *Bacteroides coprophilus DSM 18228* | 547042 | 643886197 |
| *Bacteroides coprosuis DSM 18011* | 679937 | 2503982039 |
| *Bacteroides dorei 5_1_36* | 556260 | 646206258 |
| *Bacteroides dorei DSM 17855* | 483217 | 642979370 |
| *Bacteroides eggerthii 1_2_48FAA* | 665953 | 649989912 |
| *Bacteroides eggerthii DSM 20697* | 483216 | 642979334 |
| *Bacteroides finegoldii DSM 17565* | 483215 | 642979319 |
| *Bacteroides fluxus YIT 12057* | 763034 | 651324011 |
| *Bacteroides fragilis 3_1_12* | 457424 | 645058788 |
| *Bacteroides fragilis 638R* | 862962 | 650377910 |
| *Bacteroides fragilis NCTC 9343* | 272559 | 637000024 |
| *Bacteroides fragilis YCH46* | 295405 | 637000025 |
| *Bacteroides gallinarum DSM 18171* | 1121096 | 2519899680 |
| *Bacteroides graminisolvens DSM 19988* | 1121097 | 2524614553 |
| *Bacteroides helcogenes P 36-108* | 693979 | 649633012 |
| *Bacteroides intestinalis DSM 17393* | 471870 | 642791621 |
| *Bacteroides massiliensis DSM 17679* | 1121098 | 2515154074 |
| *Bacteroides oleiciplenus YIT 12058* | 742727 | 2534681837 |
| *Bacteroides ovatus 3_8_47FAA* | 665954 | 651324012 |
| *Bacteroides ovatus ATCC 8483* | 411476 | 641380449 |
| *Bacteroides ovatus NLAE-zl-C34* | 1145612 | 2513020027 |
| *Bacteroides ovatus NLAE-zl-H304* | 1201624 | 2515154064 |

| | | |
|---|---|---|
| *Bacteroides ovatus SD CC 2a* | 702444 | 647000212 |
| *Bacteroides ovatus SD CMC 3f* | 702443 | 647000213 |
| *Bacteroides plebeius DSM 17135* | 484018 | 642979351 |
| *Bacteroides propionicifaciens DSM 19291* | 1121099 | 2517093002 |
| *Bacteroides pyogenes DSM 20611* | 1121100 | 2524614554 |
| *Bacteroides salanitronis DSM 18170* | 667015 | 649633013 |
| *Bacteroides salyersiae WAL 10008* | 1121101 | 2531839250 |
| *Bacteroides sp. 1_1_14* | 469585 | 648861000 |
| *Bacteroides sp. 1_1_30* | 457387 | 651324013 |
| *Bacteroides sp. 1_1_6* | 469586 | 646206272 |
| *Bacteroides sp. 2_1_16* | 469587 | 647533110 |
| *Bacteroides sp. 2_1_22* | 469588 | 647533111 |
| *Bacteroides sp. 2_1_33B* | 469589 | 647533112 |
| *Bacteroides sp. 2_1_56FAA* | 665938 | 651324014 |
| *Bacteroides sp. 2_1_7* | 457388 | 645058782 |
| *Bacteroides sp. 2_2_4* | 469590 | 646206266 |
| *Bacteroides sp. 20_3* | 469591 | 648861001 |
| *Bacteroides sp. 3_1_19* | 469592 | 648861002 |
| *Bacteroides sp. 3_1_23* | 457390 | 648861003 |
| *Bacteroides sp. 3_1_33FAA* | 457391 | 647533113 |
| *Bacteroides sp. 3_1_40A* | 469593 | 649989913 |
| *Bacteroides sp. 3_2_5* | 457392 | 646206273 |
| *Bacteroides sp. 4_1_36* | 457393 | 649989914 |
| *Bacteroides sp. 4_3_47FAA* | 457394 | 646206274 |
| *Bacteroides sp. 9_1_42FAA* | 457395 | 646206263 |
| *Bacteroides sp. D1* | 556258 | 646206264 |
| *Bacteroides sp. D2* | 556259 | 645951811 |
| *Bacteroides sp. D20* | 585543 | 647533114 |
| *Bacteroides sp. D22* | 585544 | 648861004 |
| *Bacteroides sp. HPS0048* | 1078089 | 2529293266 |
| *Bacteroides stercoris ATCC 43183* | 449673 | 641736196 |
| *Bacteroides thetaiotaomicron NLAE-zl-C523* | 1145805 | 2513020028 |
| *Bacteroides thetaiotaomicron NLAE-zl-H207* | 1201516 | 2515154063 |
| *Bacteroides thetaiotaomicron NLAE-zl-H492* | 1201827 | 2515154067 |
| *Bacteroides thetaiotaomicron VPI-5482* | 226186 | 637000026 |
| *Bacteroides uniformis ATCC 8492* | 411479 | 641380447 |
| *Bacteroides vulgatus ATCC 8482* | 435590 | 640753008 |
| *Bacteroides vulgatus PC510* | 702446 | 647000214 |
| *Bacteroides xylanisolvens NLAE-zl-C29* | 1145558 | 2513020025 |
| *Bacteroides xylanisolvens NLAE-zl-G310* | 1189854 | 2514885005 |
| *Bacteroides xylanisolvens NLAE-zl-G421* | 1189970 | 2514885006 |
| *Bacteroides xylanisolvens NLAE-zl-P352* | 1146212 | 2513020030 |
| *Bacteroides xylanisolvens NLAE-zl-P393* | 1146256 | 2513020031 |
| *Bacteroides xylanisolvens NLAE-zl-P727* | 1146614 | 2513020033 |

| | | |
|---|---|---|
| *Bacteroides xylanisolvens NLAE-zl-P732* | 1146620 | 2513020034 |
| *Bacteroides xylanisolvens NLAE-zl-P736* | 1146624 | 2513020035 |
| *Bacteroides xylanisolvens SD CC 1b* | 702447 | 647000215 |
| *Bacteroides xylanisolvens XB1A* | 657309 | 650377911 |
| *Bacteroidetes bacterium SCGC AAA027-G08* | 938698 | 2524023173 |
| *Bacteroidetes bacterium SCGC AAA027-N21* | 938709 | 2524023177 |
| *Balneola vulgaris DSM 17893* | 1121104 | 2515154152 |
| *Barnesiella intestinihominis YIT 11860* | 742726 | 2529292932 |
| *Barnesiella viscericola DSM 18177* | 880074 | 2509276067 |
| *Belliella baltica DSM 15883* | 232259 | 2509276058 |
| *Butyricimonas synergistica DSM 23225* | 1121129 | 2515154026 |
| *Capnocytophaga canimorsus Cc5* | 860228 | 650716019 |
| *Capnocytophaga cynodegmi DSM 19736* | 926551 | 2513237123 |
| *Capnocytophaga gingivalis ATCC 33624* | 553178 | 643886113 |
| *Capnocytophaga ochracea DSM 7271* | 521097 | 644736338 |
| *Capnocytophaga ochracea F0287* | 873517 | 649989920 |
| *Capnocytophaga sp. oral taxon 329 str. F0087* | 706436 | 651324019 |
| *Capnocytophaga sp. oral taxon 338 str. F0234* | 888059 | 651324020 |
| *Capnocytophaga sputigena ATCC 33612* | 553177 | 642979305 |
| *Cellulophaga algicola DSM 14237* | 688270 | 649633031 |
| *Cellulophaga lytica DSM 7489* | 867900 | 649633032 |
| *Chitinophaga pinensis DSM 2588* | 485918 | 644736340 |
| *Chryseobacterium caeni DSM 17710* | 1121285 | 2524614876 |
| *Chryseobacterium daeguense DSM 19388* | 1121286 | 2523533536 |
| *Chryseobacterium gregarium DSM 19109* | 1121287 | 2523533548 |
| *Chryseobacterium palustre DSM 21579* | 1121288 | 2523533629 |
| *Croceibacter atlanticus HTCC2559* | 216432 | 648028020 |
| *Cyclobacterium marinum DSM 745* | 880070 | 2506381023 |
| *Cytophaga fermentans DSM 9555* | 869213 | 2509601038 |
| *Dyadobacter fermentans DSM 18053* | 471854 | 644736356 |
| *Dysgonomonas gadei ATCC BAA-286* | 742766 | 651324026 |
| *Dysgonomonas mossii DSM 22836* | 742767 | 651324027 |
| *Echinicola pacifica DSM 19836* | 1121859 | 2515154083 |
| *Echinicola vietnamensis DSM 17526* | 926556 | 2509276020 |
| *Emticicia oligotrophica DSM 17448* | 929562 | 2506485001 |
| *Flavobacteria bacterium BAL38* | 391598 | 640612204 |
| *Flavobacteria bacterium BBFL7* | 156586 | 638341093 |
| *Flavobacteria bacterium MS024-2A* | 487796 | 643886118 |
| *Flavobacteria bacterium MS024-3C* | 487797 | 643886079 |
| *Flavobacteria bacterium MS190-1F* | 913852 | 2507262045 |
| *Flavobacteriaceae bacterium 3519-10* | 531844 | 644736368 |
| *Flavobacteriaceae bacterium JJC* | 512012 | 2515154008 |
| *Flavobacterium columnare ATCC 49512* | 1041826 | 2511231122 |
| *Flavobacterium daejeonense DSM 17708* | 1121887 | 2523533615 |

| | | |
|---|---|---|
| *Flavobacterium filum DSM 17961* | 1121889 | 2523533606 |
| *Flavobacterium gelidilacus DSM 15343* | 1121891 | 2523533607 |
| *Flavobacterium johnsoniae UW101* | 376686 | 644736369 |
| *Flavobacterium psychrophilum JIP02 86* | 402612 | 640753027 |
| *Flavobacterium rivuli DSM 21788* | 1121895 | 2519103183 |
| *Flavobacterium soli DSM 19725* | 1121897 | 2522572162 |
| *Flavobacterium sp. CF136* | 1144313 | 2513020052 |
| *Flavobacterium subsaxonicum DSM 21790* | 1121898 | 2524614667 |
| *Flavobacterium tegetincola DSM 22377* | 1121901 | 2524023138 |
| *Flectobacillus major DSM 103* | 929703 | 2509601041 |
| *Flexibacter litoralis DSM 6794* | 880071 | 2509276059 |
| *Flexithrix dorotheae DSM 6795* | 1121904 | 2515154145 |
| *Gramella echinicola DSM 19838* | 1121931 | 2524614734 |
| *Gramella forsetii KT0803* | 411154 | 639633025 |
| *Gramella portivictoriae DSM 23547* | 1121932 | 2524614725 |
| *Haliscomenobacter hydrossis DSM 1100* | 760192 | 2504756004 |
| *Joostella marina DSM 19592* | 926559 | 2509276026 |
| *Krokinobacter sp. 4H-3-7-5* | 983548 | 650716043 |
| *Lacinutrix sp. 5H-3-7-4* | 983544 | 650716044 |
| *Leadbetterella byssophila DSM 17132* | 649349 | 649633063 |
| *Maribacter sp. HTCC2170* | 313603 | 648028027 |
| *Marivirga tractuosa DSM 4126* | 643867 | 649633065 |
| *Mucilaginibacter paludis DSM 18603* | 714943 | 2506520020 |
| *Muricauda ruestringensis DSM 13258* | 886377 | 2505679007 |
| *Myroides injenensis M09-0166* | 1008457 | 2523231007 |
| *Myroides odoratimimus CCUG 10230* | 883150 | 2513237331 |
| *Myroides odoratimimus CCUG 12901* | 883152 | 2522572175 |
| *Myroides odoratimimus CCUG 3837* | 883153 | 2523231006 |
| *Myroides odoratimimus CIP 101113* | 883154 | 2522572177 |
| *Myroides odoratimimus CIP 103059* | 883155 | 2522572176 |
| *Myroides odoratus DSM 2801* | 929704 | 2506520050 |
| *Niabella aurantiaca DSM 17617* | 1122605 | 2515154175 |
| *Niabella soli DSM 19437* | 929713 | 2506783006 |
| *Niastella koreensis GR20-10* | 700598 | 2506520016 |
| *Odoribacter laneus YIT 12061* | 742817 | 2513237280 |
| *Odoribacter splanchnicus DSM 20712* | 709991 | 649633078 |
| *Olivibacter sitiensis DSM 17696* | 1122621 | 2515154027 |
| *Ornithobacterium rhinotracheale DSM 15997* | 867902 | 2509601001 |
| *Owenweeksia hongkongensis DSM 17368* | 926562 | 2508501098 |
| *Paludibacter propionicigenes WB4* | 694427 | 649633080 |
| *Parabacteroides distasonis ATCC 8503* | 435591 | 640753039 |
| *Parabacteroides johnsonii DSM 18315* | 537006 | 642979358 |
| *Parabacteroides merdae ATCC 43184* | 411477 | 640963016 |
| *Parabacteroides sp. D13* | 563193 | 646206279 |

| | | |
|---|---|---|
| *Paraprevotella clara YIT 11840* | 762968 | 2513237390 |
| *Paraprevotella xylaniphila YIT 11841* | 762982 | 651324083 |
| *Pedobacter glucosidilyticus DSM 23534* | 540906 | 2523533576 |
| *Pedobacter heparinus DSM 2366* | 485917 | 644736398 |
| *Pedobacter oryzae DSM 19973* | 468059 | 2523231054 |
| *Pedobacter saltans DSM 12145* | 762903 | 649633082 |
| *Pedobacter sp. BAL39* | 391596 | 640963036 |
| *Polaribacter irgensii 23-P* | 313594 | 638341152 |
| *Polaribacter sp. MED152* | 313598 | 638341218 |
| *Porphyromonas asaccharolytica DSM 20707* | 879243 | 2504756018 |
| *Porphyromonas asaccharolytica PR426713P-I* | 910312 | 649989985 |
| *Porphyromonas bennonis DSM 23058* | 1122971 | 2517093003 |
| *Porphyromonas endodontalis ATCC 35406* | 553175 | 643886148 |
| *Porphyromonas gingivalis ATCC 33277* | 431947 | 642555148 |
| *Porphyromonas gingivalis TDC60* | 1030843 | 650716072 |
| *Porphyromonas gingivalis W83* | 242619 | 637000209 |
| *Porphyromonas gulae DSM 15663* | 1122972 | 2518645603 |
| *Porphyromonas macacae DSM 20710* | 1122974 | 2517093006 |
| *Porphyromonas somerae DSM 23386* | 1122975 | 2515154109 |
| *Porphyromonas uenonis 60-3* | 596327 | 643886142 |
| *Porphyromonas uenonis DSM 23387* | 1122976 | 2528311143 |
| *Prevotella albensis DSM 11370* | 1122978 | 2526164514 |
| *Prevotella amnii CRIS 21A-A* | 679191 | 648276713 |
| *Prevotella amnii DSM 23384* | 1122979 | 2518645615 |
| *Prevotella baroniae DSM 16972* | 1122980 | 2526164515 |
| *Prevotella bergensis DSM 17361* | 585502 | 645951825 |
| *Prevotella bivia DSM 20514* | 868129 | 2509601036 |
| *Prevotella bivia JCVIHMP010* | 553171 | 647000292 |
| *Prevotella bryantii B14* | 752555 | 648276714 |
| *Prevotella bryantii C21a* | 1280705 | 2524614710 |
| *Prevotella buccae ATCC 33574* | 873513 | 649989986 |
| *Prevotella buccae D17* | 575611 | 647533195 |
| *Prevotella buccalis ATCC 35310* | 679190 | 647000293 |
| *Prevotella copri DSM 18205* | 537011 | 643886200 |
| *Prevotella dentalis DSM 3688* | 908937 | 2509276066 |
| *Prevotella denticola CRIS 18C-A* | 944557 | 651324088 |
| *Prevotella denticola DSM 20614* | 1122982 | 2526164516 |
| *Prevotella denticola F0289* | 767031 | 650716073 |
| *Prevotella disiens FB035-09AN* | 866771 | 648276715 |
| *Prevotella histicola F0411* | 857291 | 2513237391 |
| *Prevotella intermedia 17* | 246198 | 2501416924 |
| *Prevotella intermedia DSM 20706* | 1122984 | 2523231016 |
| *Prevotella loescheii DSM 19665* | 1122985 | 2518645604 |
| *Prevotella maculosa DSM 19339* | 1122986 | 2521172638 |

| | | |
|---|---|---|
| *Prevotella maculosa OT 289* | 999422 | 2513237350 |
| *Prevotella marshii DSM 16973* | 862515 | 648276716 |
| *Prevotella melaninogenica ATCC 25845* | 553174 | 648028051 |
| *Prevotella melaninogenica D18* | 575612 | 647533196 |
| *Prevotella micans DSM 21469* | 1122987 | 2515154159 |
| *Prevotella micans F0438* | 883158 | 2513237310 |
| *Prevotella multiformis DSM 16608* | 888743 | 649989987 |
| *Prevotella multisaccharivorax DSM 17128* | 688246 | 2503754046 |
| *Prevotella nanceiensis DSM 19126* | 1122988 | 2514885007 |
| *Prevotella nigrescens ATCC 33563* | 997352 | 651324089 |
| *Prevotella oralis ATCC 33269* | 873533 | 649989988 |
| *Prevotella oralis HGA0225* | 1203550 | 2541047008 |
| *Prevotella oris C735* | 563008 | 648861012 |
| *Prevotella oris DSM 18711* | 1122989 | 2517572237 |
| *Prevotella oris F0302* | 649760 | 645951806 |
| *Prevotella oulorum F0390* | 702438 | 2513237263 |
| *Prevotella pallens ATCC 700821* | 997353 | 651324090 |
| *Prevotella paludivivens DSM 17968* | 1122990 | 2515154072 |
| *Prevotella ruminicola 23* | 264731 | 646564560 |
| *Prevotella salivae DSM 15606* | 888832 | 649989989 |
| *Prevotella sp. AGR2160* | 1280674 | 2524614711 |
| *Prevotella sp. C561* | 563031 | 2513237323 |
| *Prevotella sp. oral taxon 299 str. F0039* | 575614 | 647533197 |
| *Prevotella sp. oral taxon 306 str. F0472* | 1095752 | 2513237374 |
| *Prevotella sp. oral taxon 317 str. F0108* | 575615 | 647533198 |
| *Prevotella sp. oral taxon 472 str. F0295* | 619693 | 647000294 |
| *Prevotella stercorea DSM 18206* | 1002367 | 2513237318 |
| *Prevotella tannerae ATCC 51259* | 626522 | 645951840 |
| *Prevotella timonensis CRIS 5C-B1* | 679189 | 647000295 |
| *Prevotella timonensis DSM 22865* | 1122992 | 2523533627 |
| *Prevotella veroralis DSM 19559* | 1122993 | 2517572238 |
| *Prevotella veroralis F0319* | 649761 | 645951836 |
| *Proteiniphilum acetatigenes DSM 18083* | 1123008 | 2518645612 |
| *Psychroflexus torquis ATCC 700755* | 313595 | 638341165 |
| *Psychroflexus tropicus DSM 15496* | 1123035 | 2518645613 |
| *Rhodothermus marinus DSM 4252* | 518766 | 646311949 |
| *Rhodothermus marinus SG0.5JP17-171* | 762569 | 2506520042 |
| *Rhodothermus marinus SG0.5JP17-172* | 762570 | 2505679078 |
| *Riemerella anatipestifer ATCC 11845 = DSM 15868* | 693978 | 2512564079 |
| *Riemerella anatipestifer RA-CH-1* | 1228997 | 2518645536 |
| *Riemerella anatipestifer RA-CH-2* | 1271752 | 2521172679 |
| *Riemerella anatipestifer RA-GD* | 992406 | 651053063 |
| *Riemerella columbina DSM 16469* | 1123058 | 2517434010 |
| *Rikenella microfusus DSM 15922* | 880526 | 2509601020 |

| | | |
|---|---|---|
| *Robiginitalea biformata HTCC2501* | 313596 | 646311950 |
| *Rudanella lutea DSM 19387* | 1089547 | 2517093013 |
| *Runella slithyformis DSM 19594* | 761193 | 2505679030 |
| *Salinibacter ruber DSM 13855* | 309807 | 637000250 |
| *Salisaeta longa DSM 21114* | 1089550 | 2517093009 |
| *Saprospira grandis str. Lewin* | 984262 | 2512564032 |
| *Sediminibacterium sp. OR43* | 1076522 | 2509887033 |
| *Sediminibacterium sp. OR53* | 925409 | 2516143025 |
| *Segetibacter koreensis DSM 18137* | 1123248 | 2516143113 |
| *Solitalea canadensis DSM 3403* | 929556 | 2509276062 |
| *Sphingobacterium sp. 21* | 743722 | 2505679080 |
| *Sphingobacterium spiritivorum ATCC 33300* | 525372 | 643886135 |
| *Sphingobacterium spiritivorum ATCC 33861* | 525373 | 645058810 |
| *Spirosoma linguale DSM 74* | 504472 | 646311954 |
| *Spirosoma luteum DSM 19990* | 1123276 | 2515154133 |
| *Spirosoma panaciterrae DSM 21099* | 1123277 | 2515154167 |
| *Spirosoma spitsbergense DSM 19989* | 1123278 | 2515154151 |
| *Tannerella forsythensis ATCC 43037* | 203275 | 2512047036 |
| *Tannerella sp. 6_1_58FAA_CT1* | 665949 | 2513237267 |
| *Thermonema rossianum DSM 10300* | 1313301 | 2523533595 |
| *Weeksella virosa DSM 16922* | 865938 | 650377986 |
| *Xylanibacter oryzae DSM 17970* | 915438 | 2509276029 |
| *Zunongwangia profunda SM A87* | 655815 | 646564591 |

## Supplementary Table 9. Cloning primers and *E. coli* expression vectors used in this study.

| Gene | Vector | Forward primer | Reverse primer |
|---|---|---|---|
| **BACOVA_03417** | pET22b[a] | CTTATGGAATTCGACGTGCCCGATATGTCAGTATGTCGCGCAG[b] | CTCCCGCTCGAGCATTGACGCATCACAGAACGAATAATCAAAGG[b] |
| **BACOVA_03419** | pET28b | CACTACCATATGAAGCAATTGCTGCCTTACCAGGAC | CGACCGCTCGAGTTACTGTACGTTCATTACAATCG |
| **BACOVA_03421** | pRSET A[c] | CGCGGATCCAATATGGAGGTGAGTGGC | CCCAAGCTTATAACCAACAGATGTTGCATACAG |
| **BACOVA_03422** | pRSET A | CGCGGATCCAACTATCAGAAAACATCGTC | CCCAAGCTTAAACTTTACAGAAATCGCTTTCCCATTATAATC |
| **BACOVA_03423** | pET21a | CGCGGATCCAATAAACAGATAACAAGTCCTGATG | CCCAAGCTTTTTGGTTATTAATACTCCACCATTGCAAGG |
| **BACOVA_03424** | pRSET A | CGCGGATCCAATCCTATTATACAAACAAAATATACTG | CCCAAGCTTTTCAAATTCCCAATAATCCAGATTGAAAAGTTTG |
| **BACOVA_03425** | pRSET A | CGCGGATCCAACTCAAAGAAAGAACAACAATTACCG | CCCAAGCTTTTTGCAAGTAGCGTATAATCCAATTGTAGTAC |
| **BACOVA_03427** | pET22b | GCGCGGGGATCCACTGACTATTTGGACAAATCT CCATATAG | GAAACGCTCGAGCCATCCCGGATTCTGGCCAAAG |
| **BACOVA_03429** | pET22b | GTACAGGGATCCTTCGAGCCGGCCATCGAGAAC | GCGGCGCTCGAGTTTCCACCCTCTATTTTGTACAAGGTTG |
| **BACOVA_03430** | pET22b | GCTGAAGGATCCGAAAGTAGTGACAATGAATTTCCTG | CATGAACTCGAGTTTTCTCACTACTTCGAGTGACTTAC |
| **BACOVA_03431** | pET21a | CGCGGATCCGACGAATATAATTGCCAGCTTC | CCCAAGCTTTTTCAATCCGTCCACTATACCTTCATAC |
| **BACOVA_03432** | pET21a | CGCGGATCCTGTTCGGGAGGGGAAG | CCCAAGCTTAAATGTCAATCTGACTGAAGTTATACTG |
| **BACOVA_03433** | pET21a | CGCGGATCCTGTATTCTTGGTTCCTGTAAAGATGAC | CCCAAGCTTTTTTCTTTCGATAACAATGTTGTCGAG |
| **BACOVA_03434** | pET21a | CGCGGATCCGCTGCGGAACCATTTGTCACTTTTATATC | CCGCTCGAGATGCGTAACCTTGCGATAAGGACTTTC |
| **BACOVA_03436** | pRSET A | CGCGGATCCAACCCGATTGTGCAAACTTGC | CCCAAGCTTTCTGTTAAACTTGTACCAATCAAAGTTAAAC |
| **BACOVA_03438** | pET21a | CGCGGATCCGACCTGAAACTGTGGTATAGTC | CCCAAGCTTTTGTTTTCCTTCGATTGTATTTACAATATAGG |
| **BACOVA_03449** | pET28b | CGGCGGAATTCATGGCTGAACAATTTGTAATATTC ACACCG | CTCCCGCTCGAGTCTACTTTCTCTCTTACTTTTCGATTC |
| **BACOVA_04385** | pRSET A | CGCGGATCCGATGGAAGTGCCTTGTG | CCCAAGCTTCTTACTAAACCCATAAGGTGGACATTC |
| **BACOVA_04386** | pET21a | CGCGGATCCAAAACAGAAAAAAGATATTTAGTTCC | CCCAAGCTTTTCATCTTTTCCCTCGATAGTAATGATTC |
| **BACOVA_04387** | pRSET A | CGCGGATCCGATGGAAGTTCGCTGAAAAAG | CCCAAGCTTTTTTGTTTTTTCAGCCTCTTTGATAATCAGG |
| **BACOVA_04390** | pET21a | CGCGGATCCATGGAGTGGTATAAAGACCCTAC | CCCAAGCTTTTCCAAATCTCCGGTAAAGTCTC |
| **BACOVA_04391** | pET22b | GTTATAGGATCCGACGATAATGGCGGAAGTTCTGTG | CATGAACTCGAGTTCTGCATCCTCTTCTTCGTCC |
| **BACOVA_04392** | pET22b | GCGCGCGAATTCTGCAGTGACTTCCTCGATTCTG | GCGGCGCTCGAGGTTAAACAAATCGGTTATTCTGTCTTC |

[a]pET vectors are from Novagen

[b]Restriction site underlined

[c]pRSET vector is from Life Technologies

## Supplementary Table 10. Primers used for site directed mutagenesis.

| Gene | Residue change | Forward primer | Reverse primer |
|---|---|---|---|
| **BACOVA_03433** | E501A | GCCTTCTGTGTCGCCGGCGCACGGTCCCATCACGG | CCGTGATGGGACCGTGCGCCGGCGACACAGAAGGC |
| **BACOVA_03433** | T370H | CACCCTGGGATAGCAAATACCATATCAATATCAATACAG | CTGTATTGATATTGATATGGTATTTGCTATCCCAGGGTG |
| **BACOVA_03438** | E361A | TCTTGGTTTCAATTATTGTGCCCAGTTTTGGGGGTATGATG | CATCATACCCCCAAAACTGGGCACAATAATTGAAACCAAGA |
| **BACOVA_03438** | D467A | TCAGTACGGCATCCGTTTCGCGCAATGCGGCTGGACAGAGG | TCTGTCCAGCCGCATTGCGCGAAACGGATGCCGTACT |

[a]Bases changed are underlined

## Supplementary Table 11. Primers used for qRT-PCR.

| Gene | Forward primer | Reverse primer |
|---|---|---|
| **BACOVA_03426** | CCCTTCCATTCAGCTCCAATTG | GCGACGGTGTAATTGATGCTGATGA |
| **BACOVA_03428** | CATCTCCATATACTCACGTTCTTTTGC | CGGAACTTTCATACGTGGACTTAGC |
| **BACOVA_04393** | ATTGTTTCGCCTGTCTGATTACTCT | ATGAACCGTAATAAAGTAAAATCGCTGGA |

**Supplementary Table 12. Crystal data statistics and refinement details**

| Data statistics* | |
|---|---|
| | BACOVA_03438 GH95 |
| Beamline | IO2 |
| Date | 22/06/13 |
| Wavelength (Å) | 0.9796 |
| Resolution (Å) | 49.31-2.81 (2.90-2.81) |
| Space group | $P2_12_12_1$ |
| Unit-cell parameters | |
| a (Å) | 53.69 |
| b (Å) | 179.1 |
| c (Å) | 205.2 |
| α, β, γ (°) | 90.0,90.0,90.0 |
| Unit-cell volume ($Å^3$) | 1973256 |
| Solvent content (%) | 53 |
| No. of measured reflections | 336786 |
| No. of independent reflections | 48329 |
| Completeness (%) | 97.6(74.5) |
| Redundancy | 7.0(3.0) |
| Rmerge (%) | 11.8(48.3) |
| <I>/<σ(I)> | 10.8(2.0) |
| **Refinement statistics*** | |
| Rwork (%) | 18.64 |
| Rfree[#] (%) | 22.80 |
| No. of non-H atoms | |
| No. of protein atoms | 12531 |
| No. of ion atoms | 13 |
| No. of ligand atoms | 24 |
| R.m.s. deviation from ideal values | |
| Bond length (Å) | 0.011 |
| Bond Angle (°) | 1.5 |
| Average B factor ($Å^2$) | |
| Protein | 43 |
| Ligand | 24 |
| Ions | 68 |
| Ramachandran plot[+], residues in allowed and most favoured regions (%) | 99.75 |

*(Values in parenthesis are for the highest resolution shell).
[#]5% of the randomly selected reflections excluded from refinement.
[+]Calculated using MOLPROBITY.

## Supplementary Table 13. Primers used to create PUL knockouts and tagged strains.

| Primer name | Sequence |
|---|---|
| **ΔPUL-XylS – left flank 1** | GCG**GTCGAC**AGGCTTCACGTTCCATTTTAGG[A] |
| **ΔPUL-XylS – left flank 2** | ATAAAGATATTCCTTCTACGGAAC |
| **ΔPUL-XylS – right flank 1** | GCG**TCTAGA**GGCTTTCCTCGCCGGAGTT |
| **ΔPUL-XylS – right flank 2** | CTGTGTGGAAAAAGGAGAAAGG |
| **ΔPUL-XylL – left flank 1** | GCG**GTCGAC**CGGTATGATTGGTATCGGTGC |
| **ΔPUL-XylL – left flank 2** | GTTTTTTTATTAACTGGCTACATTATC |
| **ΔPUL-XylL – right flank 1** | GCG**TCTAGA**AAAGATACAGTGATTGGCATTGC |
| **ΔPUL-XylL – right flank 2** | GAGGATGGGAAGAGCTTAC |
| **Tag11 F** | ATG TCG CCA ATT GTC ACT TTC TCA |
| **Tag 1 F** | ATG CCG CGG ATT TAT TGG AAG AAG |
| **Universal Reverse** | CAC AAT ATG AGC AAC AAG GAA TCC |
| **ATT1 F** | CCT TTG CAC CGC TTT CAA CG |
| **ATT1 R** | TCA ACT AAA CAT GAG ATA CTA GC |

[a]Bold are restriction sites; either SalI or XbaI for cloning into pExchange

## Supplementary References


1. Faure R, Courtin CM, Delcour JA, Dumon C, Faulds CB, Fincher GB, *et al.* A Brief and Informationally Rich Naming System for Oligosaccharide Motifs of Heteroxylans Found in Plant Cell Walls. *Aust J Chem* 2009, **62**(6)**:** 533-537.

2. Beylot MH, McKie VA, Voragen AG, Doeswijk-Voragen CH, Gilbert HJ. The Pseudomonas cellulosa glycoside hydrolase family 51 arabinofuranosidase exhibits wide substrate specificity. *Biochemical J,* **358:** 607-614 (2001)

3. McKee LS, Pena MJ, Rogowski A, Jackson A, Lewis RJ, York WS, *et al.* Introducing endo-xylanase activity into an exo-acting arabinofuranosidase that targets side chains. *Proc Natl Acad Sci U S A,* **109:** 6537-6542 (2012)

4. Nurizzo D, Nagy T, Gilbert HJ, Davies GJ. The structural basis for catalysis and specificity of the Pseudomonas cellulosa alpha-glucuronidase, GlcA67A. *Structure,* **10:** 547-556 (2002)

5. Larsbrink J, Izumi A, Ibatullin FM, Nakhai A, Gilbert HJ, Davies GJ, *et al.* Structural and enzymatic characterization of a glycoside hydrolase family 31 alpha-xylosidase from Cellvibrio japonicus involved in xyloglucan saccharification. *Biochemical J,* **436:** 567-580 (2011)

6. Katayama T, Sakuma A, Kimura T, Makimura Y, Hiratake J, Sakata K, *et al.* Molecular cloning and characterization of Bifidobacterium bifidum 1,2-alpha-L-fucosidase (AfcA), a novel inverting glycosidase (glycoside hydrolase family 95). *J Bacteriol*, **186:** 4885-4893 (2004)
